# Decitabine disrupts EBV genomic epiallele DNA methylation patterns around CTCF binding sites to increase chromatin accessibility and lytic transcription in gastric cancer

**DOI:** 10.1128/mbio.00396-23

**Published:** 2023-08-22

**Authors:** Sarah Preston-Alp, Lisa Beatrice Caruso, Chenhe Su, Kelsey Keith, Samantha S. Soldan, Davide Maestri, Jozef Madzo, Andrew Kossenkov, Giorgia Napoletani, Benjamin Gewurz, Paul M. Lieberman, Italo Tempera

**Affiliations:** 1 The Wistar Institute, Philadelphia, Pennsylvania, USA; 2 The Coriell Institute for Medical Research, Camden, New Jersey, USA; 3 Division of Infectious Diseases, Brigham & Women’s Hospital, Harvard Medical School, Boston, Massachusetts, USA; The University of North Carolina at Chapel Hill, Chapel Hill, North Carolina, USA

**Keywords:** Epstein-Barr virus, DNA methylation, CTCF, epigenetics, gastric cancer

## Abstract

Epstein-Barr virus (EBV) is associated with 10% of human gastric carcinomas, which are distinguished by a CpG island methylator phenotype. In gastric carcinoma tumors and cell lines, the EBV genome also exhibits a high degree of 5-methyl cytosine (5mC) marks, which are propagated by host DNA methyltransferases (DNMT) with each cell cycle. Therefore, we sought to determine the effect of DNMT inhibition by the small molecule Decitabine (DCB) on EBV genomic 5mC and chromatin structure in two tumor-derived gastric cancer cell lines, YCCEL1 and SNU719. Decitabine effects on EBV genomic 5mC, chromatin structure, and viral gene expression were profiled by reduced representation bisulfite sequencing, ATAC-seq, and RNA-seq, respectively. Decitabine treatment resulted in global viral genome hypomethylation and a global increase in open chromatin. The most striking finding resulted from analyzing the methylation pattern from single RRBS sequencing reads, showing that the EBV genome contains a heterogeneous pool of epigenetic states, each of which is eroded upon Decitabine treatment. We observed heterogeneous 5mC epiallele patterns around EBV genomic CTCF binding sites and lytic gene transcriptional start sites. These results highlight the importance of 5mC in maintaining EBV genomic chromatin structure and latency. Furthermore, the presence of 5mC epialleles suggests EBV^+^ gastric cancers harbor transcriptionally distinct EBV episomes, which may exert distinct functional roles in maintaining latency and driving tumorigenesis.

**IMPORTANCE:**

Epstein-Barr virus (EBV) latency is controlled by epigenetic silencing by DNA methylation [5-methyl cytosine (5mC)], histone modifications, and chromatin looping. However, how they dictate the transcriptional program in EBV-associated gastric cancers remains incompletely understood. EBV-associated gastric cancer displays a 5mC hypermethylated phenotype. A potential treatment for this cancer subtype is the DNA hypomethylating agent, which induces EBV lytic reactivation and targets hypermethylation of the cellular DNA. In this study, we identified a heterogeneous pool of EBV epialleles within two tumor-derived gastric cancer cell lines that are disrupted with a hypomethylating agent. Stochastic DNA methylation patterning at critical regulatory regions may be an underlying mechanism for spontaneous reactivation. Our results highlight the critical role of epigenetic modulation on EBV latency and life cycle, which is maintained through the interaction between 5mC and the host protein CCCTC-binding factor.

## INTRODUCTION

Epstein-Barr virus (EBV) is a gammaherpes virus that establishes latent infection in the majority (~95%) of the world’s population. Latent EBV infection, in which most of the 80 viral lytic genes are suppressed and infectious virion is not produced, is associated with several malignancies worldwide, including Burkitt lymphoma, Hodgkin lymphoma, and the epithelial cancers nasopharyngeal and gastric carcinoma (GC). Epithelial-associated cancers make up the largest cancer burden of EBV-associated malignancies (1). EBV-associated gastric cancer (EBVaGC) comprises 10% of all GC and shows distinct clinical and molecular characteristics, as compared with EBV-negative subtypes (2, 3). EBVaGC has high levels of DNA methylation and exhibits a CpG island methylator phenotype, which may be a potential target for drug treatment (4, 5).

EBV latency involves the establishment of a minimal viral transcriptional program, in which small numbers of viral genes are expressed. In GC, EBV often expresses a modified type I latency program, such as EBV nuclear antigen 1 (EBNA1), EBV-encoded small ribonucleic acids (EBERs), *Bam*HI-A rightward transcripts (BARTs), and occasionally expresses latent membrane protein 1 (LMP1), LMP2A, BARF1, and BNLF2a (6 – 10). These genes have been implicated in EBV-mediated oncogenesis. The establishment of latency is determined by epigenetic modification of the EBV genome, including 5-methyl cytosine (5mC), histone modification, and looping of the chromatin into an organized 3D structure (11 – 15). 5mC occurs on the fifth carbon of cytosine in cytosine–guanine dinucleotide pairs catalyzed by DNA methyltransferases (DNMT) (16). DNMT3a and DNM3b mediated *de novo* 5mC, whereas DNMT1 is a maintenance methyltransferase, faithfully establishing the 5mC patterning to newly synthesized daughter DNA strands. 5mC is an important epigenetic mark modulating host and viral transcription and is frequently disrupted in cancer (17 – 19). EBV genomic 5mC heterochromatic regions restrict viral gene expression to only latency genes (18, 20 – 22). Loss of 5mC leads to loss of transcriptional repression and lytic cycle reactivation (23, 24). The host protein CCCTC-binding factor (CTCF) further defines EBV genome chromatin boundaries (12, 25, 26). CTCF is a chromatin architecture protein that facilitates loop formation by bringing together enhancers and promoters, as well as acting as an insulator to demarcate euchromatic from heterochromatin. In this regard, CTCF can prevent transcriptionally repressed chromatin from encroaching into regions of active transcription. CTCF binding of the EBV genome helps maintain the chromatin structure necessary for stable latency while also allowing the virus to respond rapidly to reactivation signals (27, 28). Notably, CTCF preferentially binds to unmethylated DNA (29 – 31).

CpG methylation can be highly heterogeneous in a bulk population, resulting in epialleles with different patterns of CpG DNA methylation. To illustrate this point, consider a genomic region with 50% methylation. The methylation state could exist as (i) two equal distributions of methylation epialleles that are fully methylated or fully unmethylated or (ii) a set of epialleles that are stochastically methylated on 50% of the 5mC sites of all the epialleles. While both would be considered 50% methylated, the latter case results in greater transcription inhibition, as all epialleles recruit transcriptionally repressive 5mC binding proteins (32). Epialleles are alleles with different chromatin and 5mC states that can be inherited and are a source of phenotypic diversity (33). This example highlights the need to assess the epiallele composition about functional effects on transcription, transcription factor binding, and cellular heterogeneity.

EBV genomes persist as multicopy episomes during latent infection largely ranging from 5 to 10 copies per cell in primary GC (34). It is not known whether each viral episome in a single cell and cell population shares an identical epigenetic pattern. Little remains known about the epiallele composition of EBV episomes during latency (35), or for viral genomes more broadly. We sought to address this by using reduced representation bisulfite sequencing (RRBS) to analyze the epiallele pattern of the EBV genome. RRBS identifies site-specific methylation patterns of a single molecule, defining specific epialleles. Here we combined RRBS with chromatin immunoprecipitation sequencing (ChIP) for CTCF, Assay for Transposase-Accessible Chromatin (ATAC-seq), and RNA-seq. We analyzed the effect of the hypomethylating agent Decitabine (DCB) on EBV genome methylation patterning and its association with euchromatin domains determined by ATAC-seq. We show that EBV episomes are comprised of a heterogeneous pool of 5mC epialleles and highlight the critical role of CTCF in maintaining euchromatin regions.

## MATERIALS AND METHODS

### Cell culture of EBVaGC cell lines

YCCEL1 and SNU719 were grown in RPMI1640 media supplemented with 10% fetal bovine serum, penicillin and streptomycin (50 U/mL), and plasmocin (25 ug/L, InvivoGen). Cells were incubated at 37°C with 5% CO_2_ in a humidified chamber. Cell identity was confirmed by short tandem repeat microsatellite testing. Cells were treated with 5-Aza-2′-deoxycytidine, DCB, at 7.5 µM or an equivalent amount of dimethylsulfoxide (DMSO) at the indicated time points.

### Reduced representation bisulfite sequencing

DNA was extracted using the DNeasy Blood & Tissue Kit (Qiagen, ID 69504), according to the manufacturer’s protocol. RRBS was previously described (36). Briefly, genomic DNA was spiked with lambda phage DNA and digested with MspI restriction enzyme. Fragments were ligated with methylated adapters (NEB) to protect adapter sequences from downstream bisulfite treatment. Digested DNA was bisulfite converted using the EZ DNA Methylation Kit (Zymo). All libraries were prepared using NEBNext adapters and indexed with i7 primers. These methylation-based libraries were spiked with 25% phiX Illumina library to ensure sequence diversity and sequenced 75 bp paired-end on Illumina HiSeq 2500.

Adapter sequences were removed using TrimGalore! (https://www.bioinformatics.babraham.ac.uk/projects/trim_galore/). RRBS sequence alignment to the human gammaherpesvirus 4 (HHV4) NC_007605.1 was performed by Bismark (27). CpG counts were performed using the bismark_methylation_extractor function. Coverage thresholds for RRBS were set at greater than 10 reads per CpG site allowing for the detection of 7,538 unique CpG sites in all samples. Differential methylation analysis was performed using methylKit in R (28, 29). *P* values were adjusted for multiple testing using the sliding linear model (SLIM) method (30). Differentially methylated sites were filtered by methylation change >10% and q_adj_ <0.05, unless otherwise stated.

### RNA-seq

RNA was extracted from cell pellets using the RNeasy Mini Kit (Qiagen) according to the manufacturer’s protocol and included the on-column DNAse I treatment. Libraries were prepared using the QuantSeq (Lexogen) library preparation as previously described (37). This was generated by oligo-dT priming to produce strand-specific libraries that were sequenced on NextSeq500 (Illumina) to generate single-end 76bp reads.

### CTCF ChIP-seq preparation

Chromatin immunoprecipitation with next-generation sequencing (ChIP-seq) was performed as previously described. Briefly, 25 × 10^6^ cells per immunoprecipitation were collected and fixed with 1% formaldehyde for 15 min and then quenched with 0.25 M glycine for 5 min on ice. After 3 washes with 1× phosphate-buffered saline (PBS), pellets were resuspended in 10 mL each of a series of three lysis buffers before fragmentation in a Covaris ME220 sonicator (peak power 75, duty factor 25, cycles/burst 1,000, average power 18.8, time 720 s) to generate chromatin fragments roughly 200–500 bp in size as determined by DNA gel electrophoresis. Chromatin was centrifuged to clear debris and a 1:20 of this cleared chromatin was kept as standard input for comparison against immunoprecipitations. Chromatin was incubated by rotating at 4°C for 1 h with 8 µg of antibody against CTCF (Active Motif 61311). Chromatin–antibody complexes were precipitated using a 50 µL of Dynabeads Protein A (ThermoFisher, product No. 10001D) incubated by rotating at 4°C overnight. DNA was purified using the Promega Wizard SV Gel and PCR Clean-up Kit (product No. A9285). Libraries for sequencing were made using the NEBNext Ultra II DNA Library Prep Kit (New England Biolabs, product No. E7103) and sequenced on the NextSeq 500 (Illumina).

### ATAC-Seq

SNU719 and YCCEL1 cells were treated with DMSO or 7.5 µM DCB for the indicated time points. Then the cells were harvested, and ATAC-seq was performed in two biological replicates according to the Omni-ATAC-seq protocol with modifications. Briefly, 1 × 10^5^ cells (>95% viability) were washed in 50 mL of cold PBS, spun down at 500 × *g* at 4°C for 5 min, and resuspended in 50 mL of cold ATAC-Resuspension Buffer (RSB) (10 mM Tris-HCl, pH 7.4, 10 mM NaCl, and 3 mM MgCl_2_) containing 0.1% IGEPAL CA-630, 0.1% Tween-20, and 0.01% Digitonin. Resuspended cells were kept on ice for 3 min, then washed with 1 mL of cold ATAC-RSB containing 0.1% Tween-20 but no IGEPAL CA-630 or Digitonin. Pellet nuclei were centrifuged at 500 × *g* and 4°C for 10 min, and the supernatant was removed. The pellet was then resuspended in a 50-mL Tn5 transposase reaction mixture following the manufacturer’s protocol (Illumina Tagment DNA Enzyme and Buffer, Illumina) and incubated at 37°C for 30 min in a thermomixer with 300 rpm mixing. DNA was purified using a MinElute PCR purification kit (Qiagen) and eluted in a 10-mL Elution Buffer for library amplification. PCR amplification of fragmented DNA was done using the NEBNext HiFi PCR Master Mix (New England Biolabs) with a universal forward and sample-specific reverse oligo for sample barcoding using the following PCR conditions: initial incubations of 72°C for 5 min and 98°C for 30 s, followed by five cycles of 98°C for 10 s, 63°C for 30 s, and 72°C for 1 min. An additional number of cycles was determined for each sample through a “side” qPCR using an aliquot of the PCR as a template to determine the number of cycles needed to reach 1/3 of the max fluorescence. PCR products were run on a 1% agarose gel, regions from ~50 bp to ~1 kb were excised, and DNA was extracted using a gel extraction kit (Qiagen). Purified DNA was submitted to the Wistar Institute Genomics core facility for quality analysis and sequencing. All samples were sequenced on NextSeq500 (Illumina) to generate paired-end 2 × 42 bp reads.

### CTCF knockdown by siRNA

Cells were plated at 80% confluency the day before transfection. Either scrambled control or CTCF targeting a pool of 10 μM siRNA was transfected with Lipofectamine RNAiMAX Reagent (Invitrogen) in Opti-MEM Medium (Invitrogen), according to the manufacturer’s protocol. The final siRNA concentration was 50 nM. Cells were transfected at 24 and 48 h and collected 48 h after last transfection.

### Chromatin immunoprecipitation

For each ChIP assay, 1 × 10^7^ cells were crosslinked with 1% formaldehyde at room temperature for 10 min and the reaction was quenched with 0.125 M glycine for 5 min. Crosslinked cells were harvested and lysed with cell lysis buffer [10 mM Tris-HCl (pH 8.0), 10 mM NaCl, 0.2% NP-40] supplemented with a protease inhibitor cocktail. Nuclei were pelleted and resuspended in nuclear lysis buffer [50 mM Tris-HCl (pH 8.0), 10 mM EDTA, 1% SDS] with a protease inhibitor cocktail. Chromatin was sheared to an average size of 200–500 bp using a Covaris ME220 sonicator (peak power 75, duty factor 25, cycles/burst 1,000, average power 18.8, and time 720 s). Chromatin was diluted with ChIP dilution buffer [0.01% SDS, 1.1% Triton X-100, 1.2 mM EDTA, 16.7 mM Tris-HCl (pH 8.1), 167 mM NaCl] with protease inhibitors. For each IP, 10 μL of CTCF antibody (Active Motif) or IgG was added and rotated at 4°C overnight. Protein A Magnetic Beads (Invitrogen) were incubated for 2 h at 4°C with rotation. Beads were washed sequentially with low salt, high salt, LiCl, and TE buffer and then eluted with elution buffer (1% SDS, 0.1M NaHCO_3_). Crosslinks were reversed by 65°C incubation with proteinase K followed by RNase A treatment. DNA was purified using the Promega Wizard SV Gel and PCR Clean-Up System (Promega) according to the manufacturer’s protocol. qPCR on the ChIP and input DNA was performed using the SYBR Green Master Mix on a QuantStudio 5 Real-Time PCR System (Thermo Fisher Scientific). The relative enrichment was calculated as a percentage of input.

### Methylation-specific qPCR

Genomic DNA was extracted using the GeneJET Genomic DNA Purification Kit (Thermo Fisher Scientific) according to the manufacturer’s protocol. Bisulfite conversion of 1 μg of DNA was performed using the EZ DNA Methylation-Gold Kit (Zymo Research) following the manufacturer’s instructions. The converted DNA was eluted in 100 μL of elution buffer. A total of 15 ng of bisulfite DNA was added to each reaction. Samples were normalized to a primer sequence specific for bisulfite-converted DNA containing no CpG sites: 84 kb (Fw: AGGAGATTGATTTGGTTTATG and Rv: AACCCTACATTTTTTAATTAATTTTAC). The following primer pairs were designed to detect unmethylated CpG sites around CTCF binding sites on the EBV genome: 6.4 kb (Fw: TTTTTAGAGAGGGTAAAAGGG and Rv: CAAACTACATCACCGTAACA), 89 kb (Fw: TTGGTTAGTAGGGGTTGA and Rv: CCCTAATAAAAACTACCAACC), 91.4 kb (Fw: GGTTAAGTGGTTGGGGTAT and Rv: CCTACCAAAACCAAAAACA), and 138.8 kb (Fw: GTAGAAAATTGATAAGGATTGTG and Rv: ACTCCCACTAATTCCCAC).

### Bioinformatic analysis

Reads were mapped against the human gammaherpesvirus 4 (HHV4) NC_007605.1. For ChIP-seq data, reads were mapped to the genome assembly using BWA (38). The 10–40 kb region contains an internal repeat region (IR1) containing multiple copies of a 3-kb repeat, which makes it difficult to find uniquely mapped sequencing reads; therefore, this region is omitted from the analysis. We used MACS2[72,73] software packages to call peaks using input samples as control. deepTools (39) was used for data visualization. RNA-seq data were aligned using STAR (40). RSEM v1.2.12 software was used to estimate read counts (41). Raw counts were used to estimate the significance of differential expression differences between two experimental groups using DESeq2 (42). ATAC-seq data were aligned using bowtie2 NC_007605.1 EBV genome (43). HOMER was used to generate bigwig files and call significant peaks—style factor option (44). Peaks that passed the false discovery rate <5% threshold were considered significant. Normalized signals for significant peaks were derived from bigwig files using the bigWigAverageOverBed tool from the UCSC toolbox with the mean0 option (45). Adapter sequences were removed using TrimGalore! (https://www.bioinformatics.babraham.ac.uk/projects/trim_galore/). RRBS sequence alignment to the human gammaherpesvirus 4 (HHV4) NC_007605.1 was performed by Bismark (27). CpG counts were performed using the bismark_methylation_extractor function. Coverage thresholds for RRBS were set at greater than 10 reads per CpG site, allowing for the detection of 7538 unique CpG sites in all samples. Differential methylation analysis was performed using methylKit in R (28, 29). *P* values were adjusted for multiple testing using the SLIM method (30). Differentially methylated sites were filtered by methylation change >10% and q_adj_ <0.05, unless otherwise stated. Data have been deposited in GEO and can be accessed through the following accession number: GSE239770 and GSE234658.

### Digital droplet PCR

Multiplex digital droplet PCR (ddPCR) was performed as previously described (46). DNA was isolated from 1 × 10^6^ cells using the GeneJET Genomic DNA Purification Kit (Thermo Scientific) according to the manufacturer’s protocol. For DNA extraction from supernatant, 200 μL of supernatant was first collected and treated with DNAseI to digest cell-free DNA followed by Proteinase K treatment. A total of 500 ng of cellular DNA was digested with BamHI enzyme (10 U/µL, New England Biolabs) in a total volume of 10 µL for 1 h at 37°C. Digestion was diluted 1:20 in nuclease-free water. An amount of 10 µL of diluted DNA digest was mixed with 12.5 µL of 2× digital PCR supermix for probes (No dUTP) (Bio-Rad), 1.25 µL 20× FAM primers, and 1.25 µL VIC primers for each reaction. The FAM primers sequence for EBV *Lmp1* was Fw (5′−3′) AAGGTCAAAGAACAAGGCCAAG, Rv (5′−3′) GCATCGGAGTCGGTGG, and FAM - AGCGTGTCCCCGTGGAGG. Host control primer sequence for Ribonuclease P protein subunit 20 (Rpp30) was Fw (5′−3′) GATTTGGACCTGCGAGCG, Rv (5′−3′) GCGGCTGTCTCCACAAGT, and probe VIC-CTGACCTGAAGGCTCT. 20× primers contain 18 µM PCR primers and 5 µM probes for a final PCR concentration of 900 nM PCR primers and 250 nM probes. Each sample was run in duplicate. The ddPCR plate was sealed with a foil heat seal using the PX1 PCR Plate Sealer (Bio-Rad) at 180°C for 5 s. The plate was vortexed and spun down at 1,000 rpm for 1 min. Droplets were generated using the QX200 Droplet Digital PCR System (Bio-Rad) and the transfer of emulsified samples to a PCR plate was performed, according to the manufacturer’s instructions. The PCR plate containing emulsified droplets was sealed with a foil heat seal. PCRs were performed on the C1000 Touch Therma Cycler (Bio-Rad). The cycling protocol included an enzyme activation step at 95°C for 10 min and cycled 40 times between a denaturing step at 94°C for 30 s and an annealing and extension step at 60°C for 1 min, finally one enzyme deactivation step was performed at 98°C for 10 min. The ramp rate between these steps is set at 2 °C/s. Droplets were then counted using QX200 Droplet Reader (Bio-Rad). The absolute quantity of DNA per sample was determined using the QuantaSoft software.

## RESULTS

### The hypomethylating agents target CpG sites in EBVaGC

**Fig 1:**
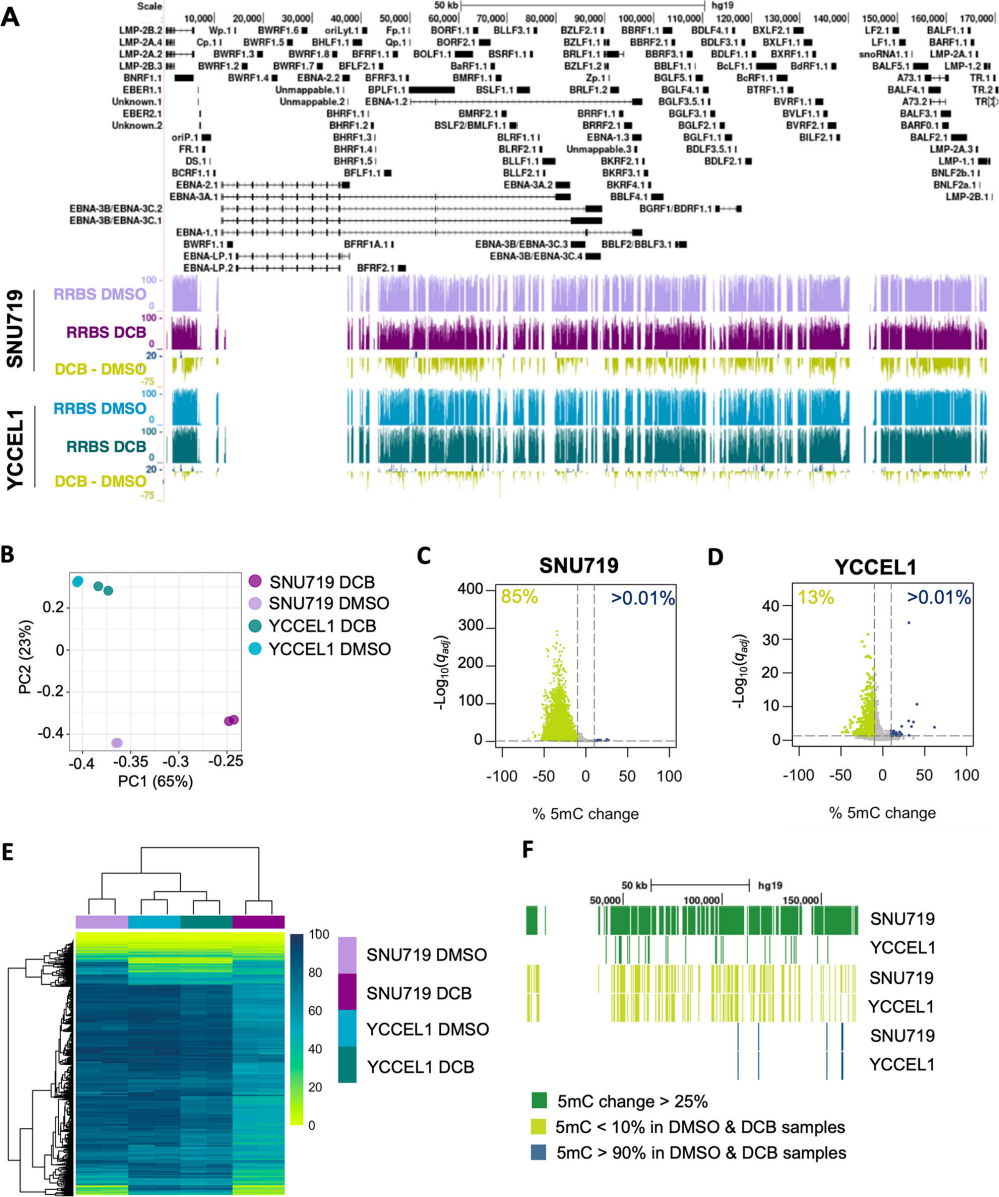
DCB induces global hypomethylation of the EBV episome. (**A**) EBV genomic tracks showing 5mC before and after DCB treatment in the SNU719 and YCCEL1 cell lines. Representative tracks are an average of two biological replicates. (**B**) PCA plot based on the 5mC levels of the EBV episome. (**C**) Volcano plot showing the significance of the percentage change in 5mC after DCB treatment for SNU719 or (D) YCCEL1 cell lines. (**E**) Heatmap showing the level of methylation from 0% to 100% (yellow to blue, respectively) for all samples. (**F**) Genomic location of CpG sites with the greatest changes (<25% 5mC change, top green), CpG sites hypomethylated in all samples (<10% 5mC, middle yellow), and CpG sites hypermethylated in all samples (>90% 5mC, bottom blue).

EBV genomes are hypermethylated in EBVaGC with a type II latency pattern. Hypomethylating agents are a potential therapy for EBVaGC as a tool to reactivate. To define the effects of hypomethylating agents on EBV genomic 5mC patterning in GC, we treated two EBVaGC cell lines, SNU719 and YCCEL1, with 5′aza-2′-deoxycytidine or DCB for 72 h. We chose this time point as these cell lines divide approximately once every 72 h, allowing DNMT inhibition effects on viral genomic 5mC to become evident. SNU719 was derived from a primary EBV +GC tumor, whereas YCCEL1 was obtained from a metastatic tumor (47, 48). We then analyzed 5mC site-specific changes by RRBS. RRBS is an efficient high-throughput sequencing technique that allows for the detection of site-specific changes in the 5mC pattern. This approach highlighted an EBV genome-wide hypomethylating effect on DCB treatment of SNU719, and to a lesser extent YCCEL1 (Fig. 1A). It should be noted that while the EBV genome is portrayed linearly, it is a circularized genome. Principal component analysis of 5mC modification demonstrated a clear separation between SNU719 DMSO and DCB-treated samples, and to a lesser extent also for YCCEL1 (Fig. 1B). DCB treatment of SNU719 resulted in hypomethylation (differential 5mC > 10%) for 85% (*n* = 6344) of EBV genomic CpG sites (Fig. 1C). Less than 0.01% (*n* = 39) of CpG sites were found to be hypermethylated by DCB treatment. In YCCEL1, 13% of CpG sites (*n* = 1008) were hypomethylated and less than 0.01% (*n* = 34) were hypermethylated by DCB (Fig. 1D). A total of 7538 CpG sites were detected in all samples across the EBV genome, many were found to be highly methylated and susceptible to DCB treatment (Fig. 1E). A subset of EBV genomic 5mC sites had low levels of 5mC in both cell lines and were unaffected by treatment. Furthermore, we observed several viral genomic CpG sites with low levels of methylation only in SNU719. Interestingly, high levels of 5mC marks remained unaffected by DCB treatment in both cell lines: SNU719 (*n* = 56, 0.75%) and YCCEL1 (*n* = 2412, 32%) (Fig. 1F). The presence of these highly methylated CpG sites resistant to hypomethylation suggests that there is a minimal contribution from newly synthesized EBV genomes, which should be completely hypomethylated. Overall, these results indicated that the global 5mC levels and response to DCB were conserved between the two cell lines (Fig. S1).

### EBV genome hypomethylation is associated with increased chromatin accessibility in EBVaGC

**Fig 2:**
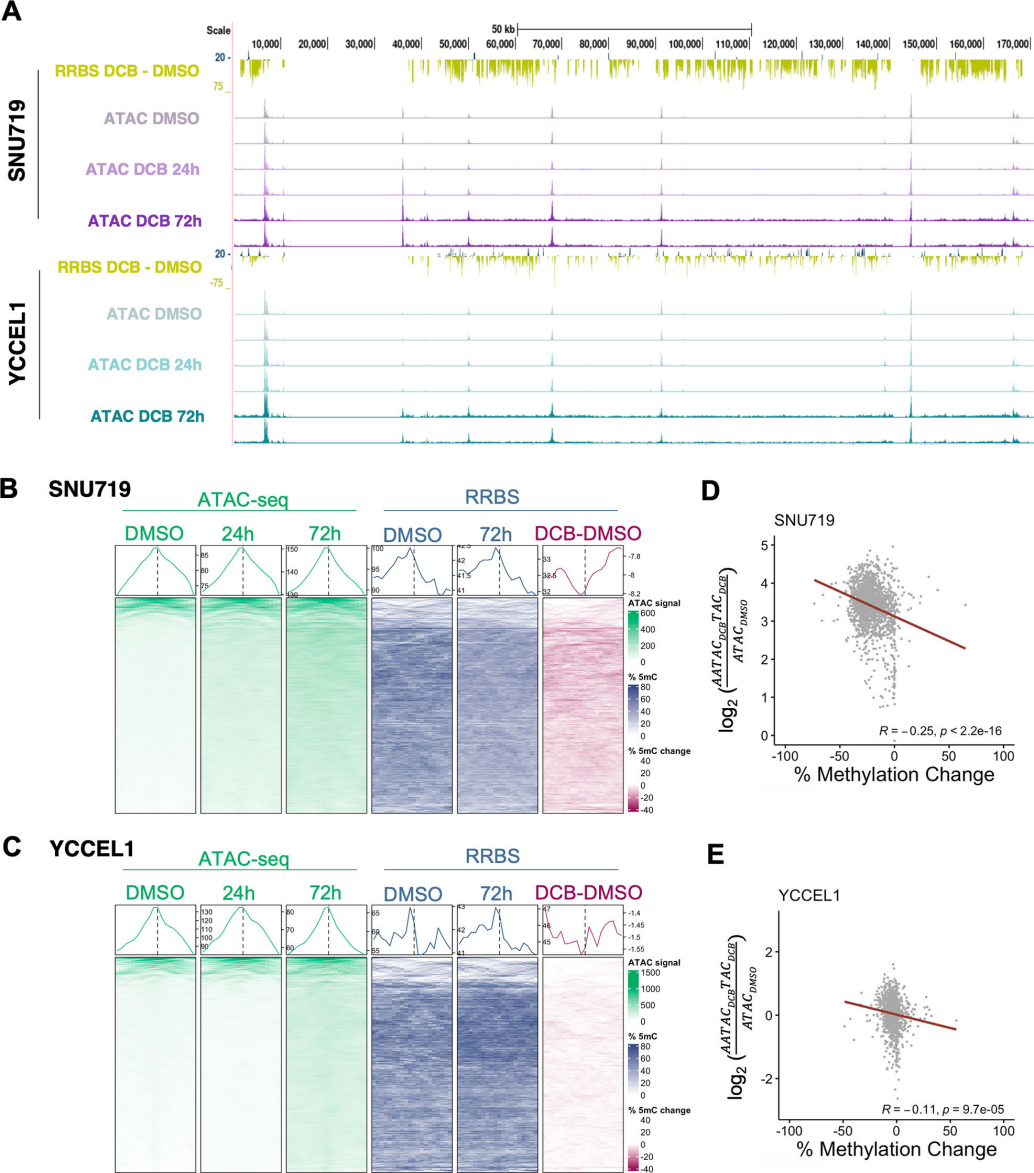
DCB-induced hypomethylation correlates with euchromatin of the EBV episome. (**A**) EBV genomic tracks showing changes in 5mC after DCB treatment (top), with ATAC-seq signal peaks for DMSO, 24- and 72-h treated DCB for SNU719 and YCCEL1. Heatmaps centered on 1,000 bp region surrounding the ATAC-seq peak (DMSO, 24-h, and 72-h DCB treatment) and RRBS data (DMSO and 72-h DCB treatment) for (B) SNU719 and (C) YCCEL1. Heatmaps are in descending order with the strongest ATAC peak at the top. A scatterplot shows the correlation between the change in methylation and the log_2_(fold change) in the ATAC-seq signal after DCB treatment. Linear regression line is shown in red with Pearson’s correlation coefficient and *P* value for (D) SNU719 and (E) YCCEL1.

Since 5mC is associated with heterochromatin and silenced gene expression, we sought to define DCB-induced hypomethylation effects on EBV chromatin accessibility. Therefore, we performed Assay for Transposase-Accessible Chromatin (ATAC-seq) to identify regions of open chromatin on control vs DCB-treated cells. EBV genomes from control cells exhibited approximately seven distinct open chromatin peak regions: 6.5 kb/EBER1, 36 kb, 50 kb/Qp, 68 kb, 91 kb/Zp, 144.5 kb, and 166.5 kb/LMP1. These peaks were conserved in SNU719 and YCCEL1 and remained at both 24 and 72 h of DCB treatment (Fig. 2A). By contrast, DCB treatment resulted in a general opening of EBV chromatin, defined by globally increased ATAC-seq signal by 72 h of treatment. Several smaller accessible peaks evident in control cell genomes became more pronounced after 72 h of DCB treatment: 10.5 kb/Cp, 41 kb/oriLyt, and 139 kb (Fig. 2A). In SNU719, we observed several strong ATAC-seq peaks across all treatment conditions in areas of low methylation (Fig. 2B). Areas with minimal ATAC-seq peaks were found in regions with high 5mC. After treatment, these areas show an opening in chromatin with increased ATAC signal and loss of 5mC. Similar effects were observed in YCCEL1 (Fig. 2C). Negative correlation between loss of 5mC and gain of ATAC signal was observed in both cell lines after 72 h of DCB treatment (Fig. 2D and E). Our data correlate DCB-induced hypomethylation with a pronounced effect on relaxing heterochromatin across the EBV genome.

### CTCF occupancy defines areas of EBV genome open chromatin in EBVaGC

**Fig 3:**
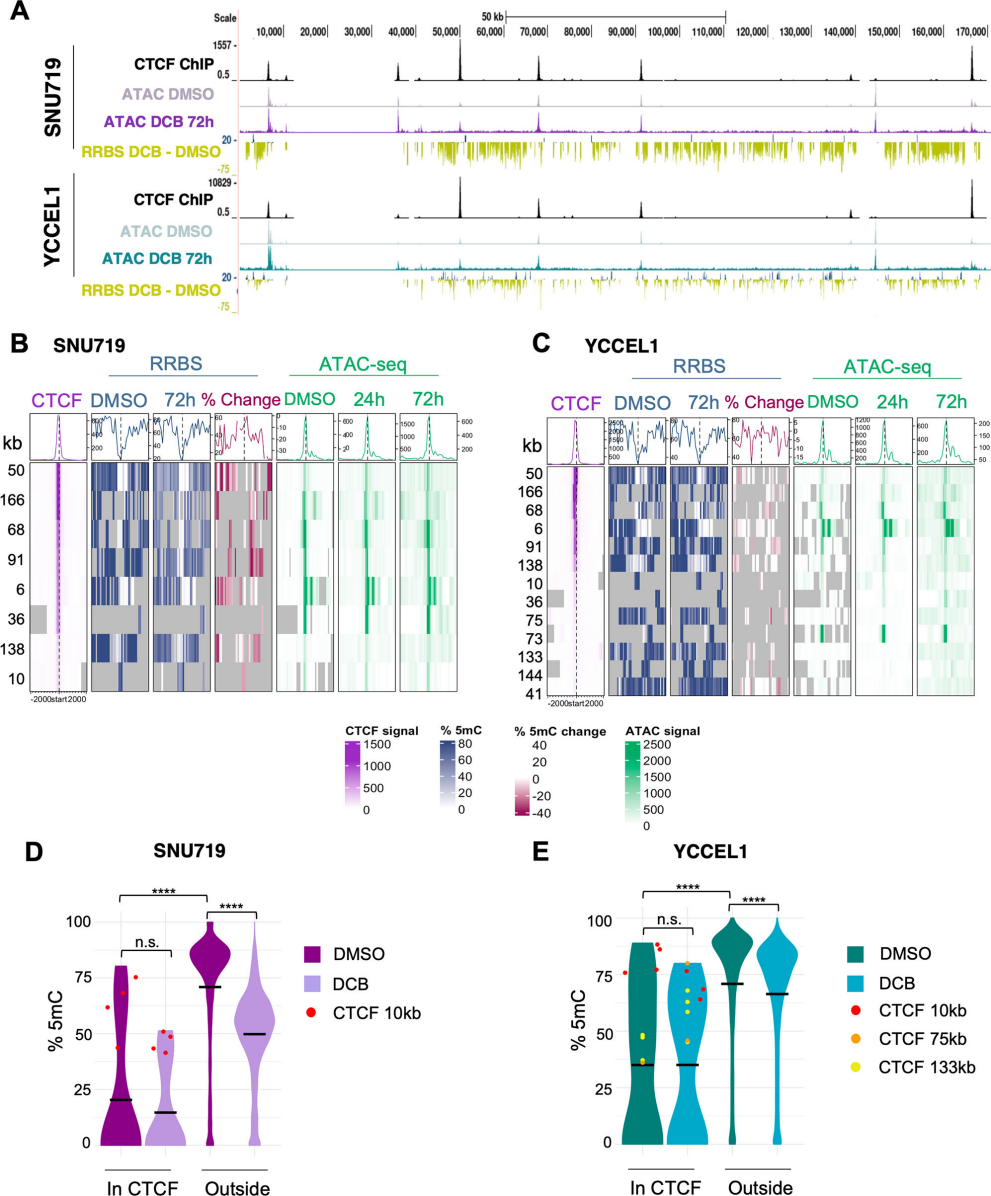
DCB-induced hypomethylation correlates with euchromatin at CTCF binding sites. (**A**) EBV genomic tracks for CTCF binding sites, ATAC-seq, and changes in 5mC after DCB treatment. Heatmaps of 2,000 bp regions upstream and downstream of the CTCF peak center for CTCF ChIP-seq, ATAC-seq DMSO, ATAC-seq 24 and 72 h DCB, and RRBS 5mC DMSO and 72 h DCB for (B) SNU719 and (C) YCCEL1. Violin plots show the percentage of 5mC of CpG sites in CTCF binding regions or outside before and after treatment for (D) SNU719 and (E) YCCEL1. The black bar indicates the mean. CpG sites for heavily methylated CTCF binding regions are highlighted: 10 kb (red), 75 kb (orange), and 133 kb (yellow).

Since CTCF is an important chromatin architecture protein that regulates host and viral transcription, we next examined how DCB treatment affects EBV chromatin and 5mC marks at CTCF binding sites. To define the CTCF binding landscape in SNU719 and YCCEL1, we performed ChIP-seq. Our analysis revealed 10 EBV genomic CTCF binding sites in SNU719 and 13 in YCCEL1 (Fig. 3A). Common prominent CTCF binding sites occurred at 6 and 10 kb around OriP, 50 kb viral Q promoter, 68 kb within *EBNA1*, 91 kb within the *BZLF1* promoter, and 170 kb at the LMP1 promoter. The 36 kb CTCF site was present in SNU719 but was diminished in YCCEL1. Strikingly, CTCF binding sites overlapped almost exclusively with prominent ATAC-seq signals. CpG sites were not methylated in or around strong CTCF binding sites in either SNU719 or YCCEL1, even before DCB treatment (Fig. 2B and C). Some CTCF binding sites, such as the 10 kb, show weak CTCF binding, lower ATAC-seq signal, and higher levels of 5mC. In SNU719, CpG sites in CTCF binding regions were significantly less methylated than CpG sites outside of CTCF binding sites (Fig. 2D). This contrasted with EBV genomic CpG sites outside of CTCF binding sites, which were highly methylated in control cells and significantly hypomethylated by DCB treatment. To determine the effect of CTCF binding on the 5mC of these regions, we performed CTCF knockdown in the YCCEL1 cells and found a significant reduction in the unmethylated CpG sites around the EBER1 CTCF binding site (Fig. S2). Our data highlight the important functional role of CTCF as an insulator and protector of important transcriptional regulatory regions of the EBV genome from 5mC.

### Decitabine disrupts DNA methylation epialleles around CTCF binding sites in EBVaGC

**Fig 4:**
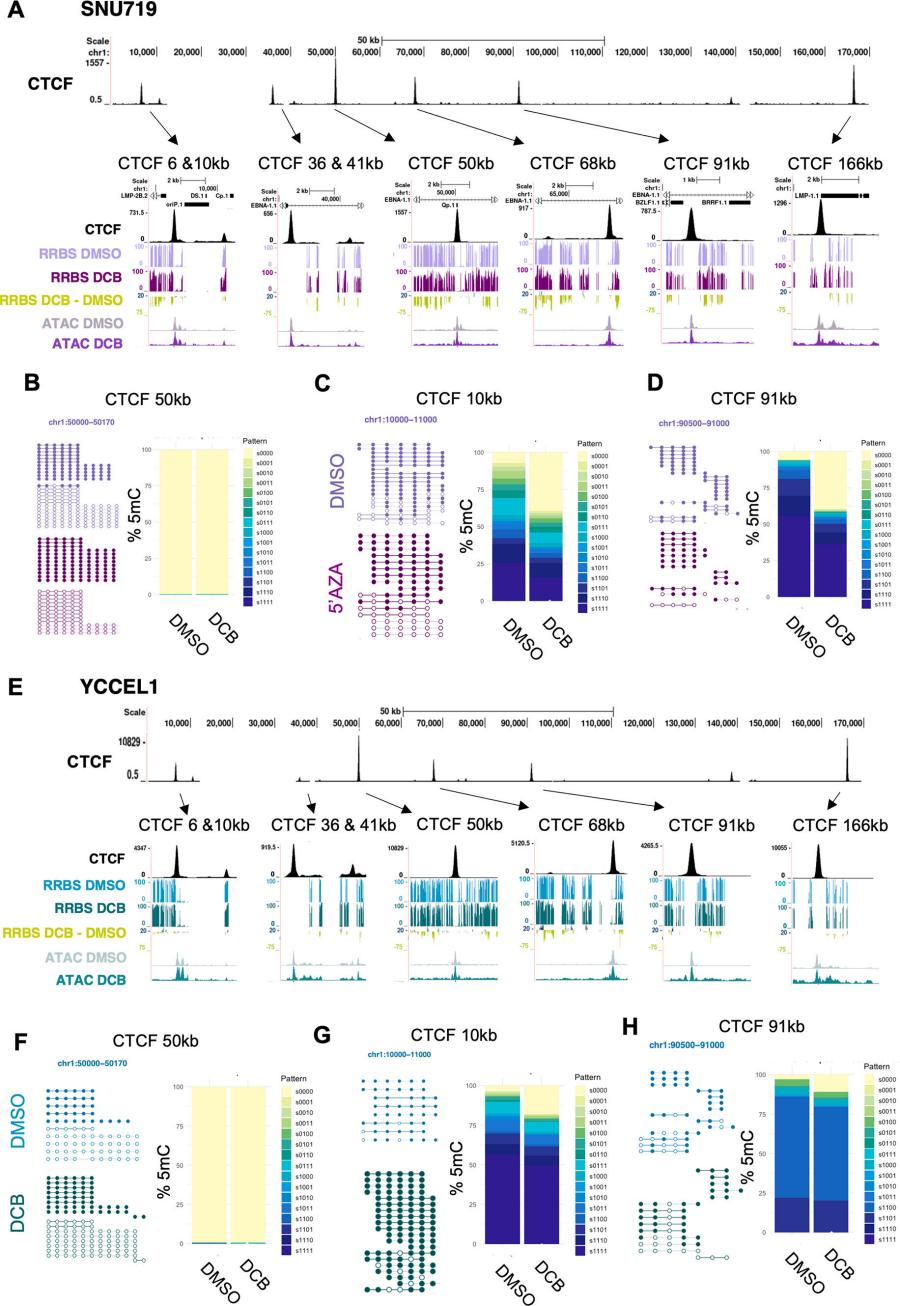
DCB disrupts 5mC patterning around CTCF binding sites. (**A**) EBV genomic track for CTCF-ChIPseq for SNU719. 5mC epiallele patterns (representative sample) around (B) CTCF 10 kb, (C) CTCF 50 kb, and (D) CTCF91kb binding sites for DMSO and DCB treatment with a stacked bar plot quantify the percentage of distribution of epialleles. Methylation patterning is represented by 1 for a methylated CpG or 0 for an unmethylated CpG. Patterns were quantified for groups of four consecutive CpG sites resulting in 16 possible methylation combinations. (**E**) EBV genomic track for CTCF-ChIPseq for YCCEL1 5mC epiallele patterns (representative sample) around (F) CTCF 10 kb, (G) CTCF 50 kb, and (H) CTCF 91 kb binding sites for DMSO and DCB treatment with a stacked bar plot quantifying the percentage of distribution of epialleles.

To further investigate 5mC patterning at EBV genomic CTCF binding sites, we cross-compared CTCF ChIP-seq with RRBS data from control vs DCB-treated cells. EBV genome CTCF binding sites sat in demethylated pockets even in control cells, tightly flanked by high levels of 5mC, such as observed at positions 6 kb/*OriP*, 41 kb/, 50 kb/Qp, 68 kb/*BMRF1*, 91/*BRLF1,* and 166 kb/*LMP1* on the EBV genomic map (Fig. 4A). At the CTCF-bound site at position 36 kb from the left of the EBV genome map, CTCF instead bound to a region lacking surrounding 5mC. The strongest CTCF binding peak was found at the 50 kb EBV genome position, located at the Q promoter (Qp), which drives the expression of the latency gene, EBNA1. 5mC patterning was quantified for groups of four sequential CpG sites either methylated or unmethylated resulting in a total of 16 possible methylation patterns. 5mC patterning located in this region (50,000–50,170 kb) exhibited two distinct forms, a completely methylated (s1111) minor fraction where sequential CpG sites were methylated or a fully unmethylated (s0000) majority (Fig. 4B). In addition to Qp, we found a minor CTCF binding site at the C promoter (Cp), located at ~10 kb, which is one of the only CTCF binding sites containing methylated CpG sites. Notably, Cp is silenced in EBVaGC to block the expression of the latency III program. The majority of EBV genomic 5mC epialleles found between 10 and 11 kb region were methylated, either fully methylated or containing variable 5mC patterning (Fig. 4C). Only a minority were fully unmethylated and this increased after 72-h DCB treatment. The 91-kb CTCF binding site, located upstream of the immediate early gene BZLF1 promoter, had few methylated CpG sites. The 500 bp upstream of this CTCF binding site instead contained highly methylated epialleles (Fig. 4D). DCB treatment reduced the proportion of epialleles containing heterogeneous 5mC patterning in this region.

Many EBV genomic CTCF binding sites likewise exhibited high levels of flanking 5mC signals in YCCEL1 (Fig. 4E), suggesting that this phenotype may be conserved in EBVaGC. Interestingly, the SNU719 CTCF peak present at the EBV genomic 36 kb region was not found in YCCEL1, and this region exhibited a high degree of methylation in YCCEL1. The CTCF 50 kb peak at Qp is also the most pronounced in YCCEL1, where we again observed a low level of CpG methylation within the CTCF binding region (Fig. 4F). The smaller CTCF 10 kb peak at Cp contains mostly methylated epialleles that were hypomethylated by DCB treatment (Fig. 4G). In addition, CTCF peaks at 6 kb and 41 kb contained a heterogeneous methylated epiallele pattern (Fig. S4). Interestingly, the BZLF1 promoter region upstream of the CTCF 91 kb peak showed predominantly heterogeneous 5mC patterning (Fig. 4H), in contrast to SNU719 epialleles, most of which were fully methylated. Overall, our data suggest that EBV genomic CTCF binding occurs in hypomethylated regions in EBVaGC.

### Decitabine-induced hypomethylation causes EBV lytic reactivation in EBVaGC

**Fig 5:**
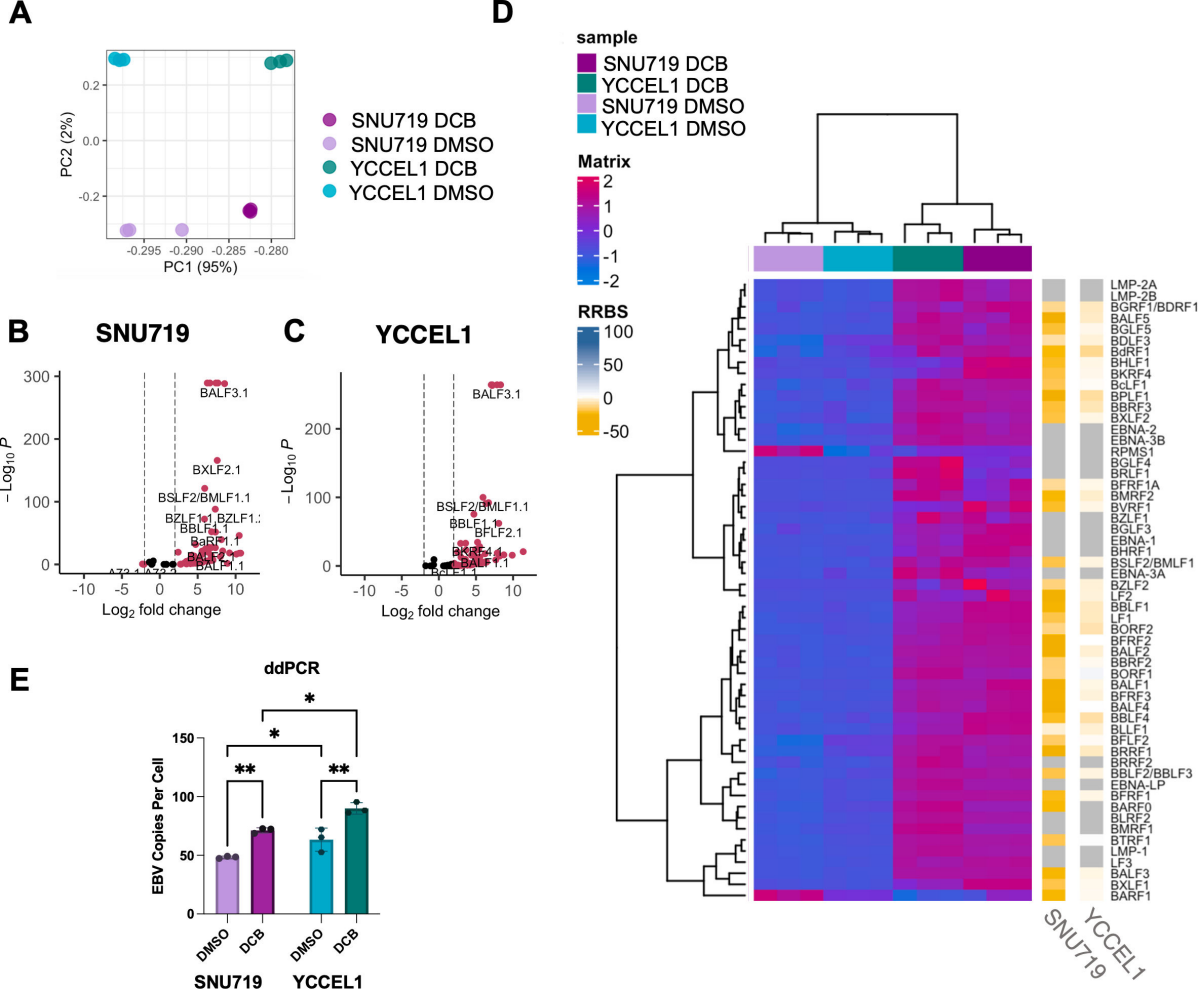
DCB induces massive expression of the EBV transcriptome and increased EBV lytic transcription. (**A**) PCA plot of RNA-seq data for SNU719 and YCCEL1 EBV transcriptome. Volcano plot showing significantly changed transcripts for (B) SNU719 and (C) YCCEL1. (**D**) A heatmap showing the transcriptional changes in the significantly changed genes in both SNU719 and YCCEL1. The change promoter 5mC (500 bp upstream of TSS) was determined by RRBS for both cell lines, where gray indicates no detectable CpG sites in the region. (**E**) Digital droplet PCR measuring LMP1 of the EBV genome after 72 h of DCB treatment.

We next used Quant-seq to define DCB hypomethylation effects on EBV transcription in YCCEL1 and SNU719. Principal component analysis (PCA) highlighted significant differences between each group, with separation between the DMSO- and DCB-treated groups along the PC1 axis explaining 95% of the mRNA variability so that the samples clustering closer together had a more similar transcriptional profile (Fig. 5A). In both SNU719 and YCCEL1, DCB treatment caused a significant increase in the mRNA abundances of many EBV genes (Fig. 5B and C), particularly the viral lytic genes including BZLF1 (Fig. S4). Notably, DCB significantly hypomethylated EBV lytic gene promoters (Fig. 5D), suggesting that 5mC marks are a major mechanism by which latency is maintained in EBVaGC. DCB also upregulated transcripts encoding the EBNA1, LMP1, and LMP2A EBV latency genes, despite the absence of 5mC marks at their promoters in untreated cells, consistent with the EBV lytic cycle driving their increased expression (49 – 51). The EBV-encoded long non-coding RNA RPMS1 was one of the few EBV transcripts that exhibited cell-specific differences, with DCB-induced expression only in SNU719. Unexpectedly, the EBV BARF1 transcript was the only lytic cycle gene downregulated by DCB treatment. In line with the observed transcriptional changes, DCB treatment increased EBV genome copy number per cell, suggestive of productive EBV lytic replication (Fig. 5E). In addition, titrating DCB concentration shows a positive correlation between DCB concentration and EBV genome copy until toxicity is reached at high concentrations above 7.5 μM (Fig. S3). Furthermore, to determine active virion production from abortive lytic replication, we determined the EBV copy number in the supernatant after DCB treatment.

### Decitabine disrupts DNA methylation epiallele patterning around EBV transcriptional start sites

**Fig 6:**
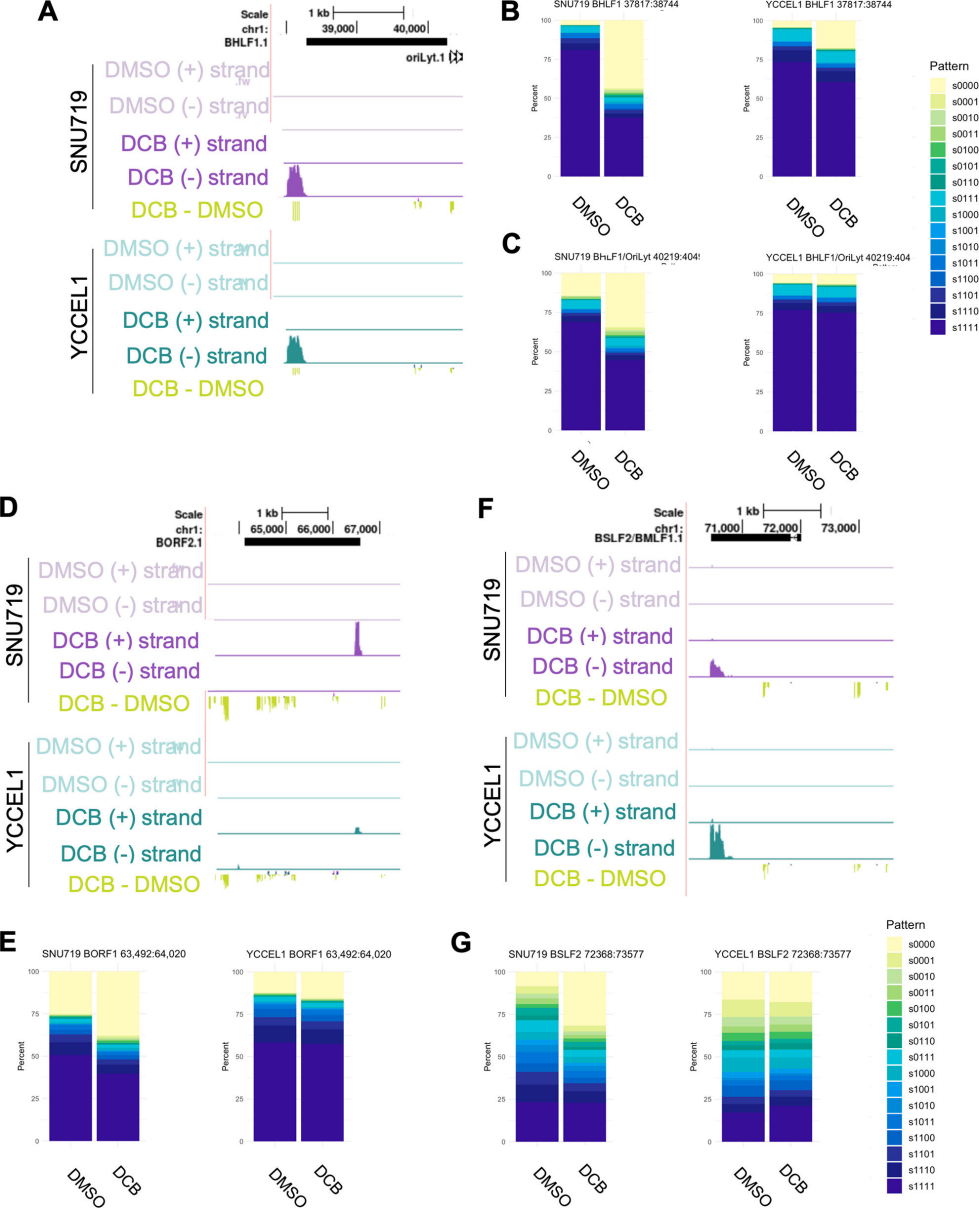
Hypomethylation and loss of 5mC patterning are correlated with increased transcription. (**A**) EBV genomic Quant-seq tracks for forward (+) and reverse (−) transcripts, and 5mC change (RRBS DCB–DMSO) in SNU719 before and after DCB treatment. The region surrounds the BHLF1 (− strand) transcript. (**B**) Stacked bar plot of the epiallele quantification for two regions upstream and downstream of BHLF1. (**C**) EBV genomic tracks for BORF1 (+ strand) transcript. (**D**) Epiallele quantification of a CpG dense region upstream of the *BORF1* transcription start site. (**E**) EBV genomic plots for BSLF2 (− strand) transcript. (**F**) Epiallele quantification of CpG sites in a region upstream of the *BSLF2* transcriptional start site.

DCB caused extensive hypomethylation of two EBV genomic regions, located at the end of the *BHLF1* coding region and within the origin of lytic replication, *oriLyt* in the 38–45 kb region (Fig. 6A). BHLF1 was recently implicated in the control of EBV programs, including viral latency in B cells, although its roles in epithelial cells are little studied (52). *BHLF1* was not expressed in control GC cells, and the majority of 5mC epialleles were methylated. By contrast, DCB increased *BHLF1* locus unmethylated epialleles and upregulated *BHLF1* expression in both SNU719 and YCCEL1 (Fig. 6B). Likewise, *oriLyt* is implicated in a reversal of EBV latency in B cells (27, 53). *OriLyt* region epialleles were methylated in control cells, although SNU719 had a larger proportion of unmethylated alleles in this region (Fig. 6C). Nonetheless, DCB caused *oriLyt* epiallele hypomethylation to a greater extent in SNU719. DCB treatment had variable effects on EBV early genes. For instance, the ribonucleotide reductase encoding EBV early lytic cycle gene *BORF2* was upregulated by DCB, with concomitant loss of 5mC throughout the *BORF2* coding region (Fig. 6D). Many *BORF2* promoter epialleles were either fully methylated or unmethylated, in contrast to the epialleles found in the EBV early lytic gene *BSLF2/BMLF2* promoter region (Fig. 6E). Nonetheless, the *BSLF2/BMLF2* transcript was upregulated by DCB treatment, with concomitant loss of 5mC marks at the *BSLF2/BMLF2* locus (Fig. 6F); however, epiallele patterning was more heterogeneous in both SNU719 and YCCEL1 (Fig. 6G). Our data highlight widespread DCB de-repression of the EBV genome in EBVaGC and show that while many epiallele compositions are conserved between the two cell lines, there are nonetheless metastable epialleles.

## DISCUSSION

DNA methylation, histone modifications, and chromatin looping each shape gene expression profiles, although the extent to which they control the EBV genome program in gastric carcinoma has remained incompletely understood. Here, we observed widespread effects of DNA hypomethylation on the viral epigenome and in two tumor-derived EBVaGC cell line models, YCCEL1 and SNU719. Near global hypomethylation triggered increased EBV genome accessibility and lytic reactivation. Our multi-omic analyses highlight a major CTCF insulating role in the control of the EBV genome in EBaGC. Areas of open chromatin corresponded almost identically to CTCF binding sites. Many of these CTCF binding sites sit in an unmethylated region, flanked by regions of hypermethylation, including key viral control elements such as the immediate early gene BZLF1 promoter and the EBNA1 Q promoter. It should be noted that newly synthesized EBV genomes are unmethylated raising the possibility that the hypomethylation of the EBV genome could arise from increased EBV replication during lytic reactivation. However, we detect several regions resistant to DNA hypomethylation after DCB treatment, suggesting that we are detecting chromatinized EBV genomes contained within the cells.

CTCF typically binds to hypomethylated DNA elements (29, 30), although the extent to which this occurs on the EBV genome has remained incompletely understood, particularly in EBVaGC. While we generally observed low levels of DNA methylation at CTCF-occupied regions, as expected, we also observed CTCF binding peaks to occur at regions that contain only a small subset of unmethylated epialleles, such as viral C promoter, *EBER*, *LMP1/LMP2*, *oriLyt*, and *BZLF1*. CTCF binding can be affected by 5mC (23). In line with this, we observe weaker CTCF binding peaks at regions that contain only a small subset of unmethylated epialleles such as the CTCF 10 kb site at Cp. Our data suggest that only a fraction of EBV genomes bind this site due to the smaller portion of unmethylated epialleles, resulting in a smaller CTCF peak. In response to DCB hypomethylation, viral euchromatin increases around these sites, which may act as transcriptional hubs in lytic reactivation (54). Based on these observations, we are tempted to speculate that CTCF sites evolved at key control elements across the viral genome to suit the needs of EBV to preserve critical regulatory regions from epigenetic silencing through DNA methylation. Such a mechanism would allow the minimal transcription activity necessary for establishing latent infection. Consistent with this hypothesis, EBV episomes carrying mutations at CTCF binding sites usually display altered gene expression patterns and an altered epigenetic landscape (55). For example, EBV episomes carrying mutations that ablate CTCF occupancy at the Qp promoter show increased levels of DNA methylation over time, eventually resulting in Qp silencing (13). More experiments are needed to fully understand the precise role of CTCF binding across the EBV genome; nevertheless, our results indicated the essential role of CTCF binding in organizing the epigenetic landscape of the EBV episome during latent infection.

Host enzyme methylation of the viral genome is thought to be a protective mechanism that silences expression from foreign DNA elements. However, EBV has evolved to circumvent this inhibitory effect by encoding a 5mC-binding transcription factor, Zta encoded by the *BZLF1* gene (56 – 59). Indeed, we observed that many EBV lytic genes contain completely methylated epialleles, such as *BHLF1* and *BSLF2*. Interestingly, we observed a heterogeneous pool of epialleles in the *BZLF1* promoter, which corresponds to a CTCF binding site at 91 kb. We speculate that heterogeneous 5mC patterning at the BZLF1 promoter may be an underlying mechanism for spontaneous reactivation. Zta works in concert with Rta, and Zta alone may function primarily to facilitate DNA replication at *OriLyt* (60). In line with this, we observed more stochastic 5mC patterning at the 91 kb region in YCCEL1, which correlated with higher EBV genome copy number, both before and after DCB treatment, relative to SNU719. Overall, there was an increase in lytic transcription which correlated with hypomethylation of many gene promoters. However, it is unclear what the contribution of hypomethylation of the host genome is on increased viral expression.

The Qp promoter is transcriptionally active in EBVaGC, where it is hypomethylated and drives the expression of EBNA1. While we found many 5mC epialleles within the Qp region to be completely unmethylated, there was also a subset that was completely methylated. Given also that EBVaGC contains many copies of the EBV episome, our data suggest that some gastric cancer EBV episomes are silenced at the Q promoter. They may function using another promoter, such as Cp, to drive EBNA1; however, this promoter contained mostly hypermethylated epialleles and was previously shown to be transcriptionally silent. An alternative and more compelling possibility is that there is a transcriptionally dead EBV genomic pool, and that these “zombie” episomes may survive passively due to other transcriptionally functional episomes present. Furthermore, they may serve as a template for replication or provide another unknown function that requires further investigation. How these heterogeneous EBV populations are distributed among tumor cells remains to be characterized. Technological advances, including the ability to detect EBV genomic 5mC patterning at the single-cell level, will undoubtedly yield further insights. Developing sequencing technologies, which offer the promise to analyze which EBV genomic epialleles interact, will provide further insights into the interplay of viral genome 5mC, CTCF, and latency states.

Altogether, these results highlight the critical role of epigenetic modulation in controlling the EBV life cycle. The critical interaction between 5mC and CTCF is disrupted after DCB treatment along with relaxation of the EBV genome and increased lytic transcription. We identified a heterogeneous pool of EBV episomes which may contribute to the specific expression pattern found in Type II latency of gastric cancer. It is possible that “zombie” EBV episomes may be related to the heterogeneous cellular phenotypes displayed among the tumor cells. Since other herpesviruses are epigenetically silenced and establish latency, it will be interesting to cross-compare how 5mC patterning, including epiallele formation and CTCF occupancy, shapes their epigenomes and latency programs.

## Data Availability

Data have been deposited in GEO and can be accessed through the following accession numbers: GSE239770 and GSE234658.

## SUPPLEMENTAL MATERIAL

Fig. S1 mbio.00396-23-s0001.tif Conserved response to DCB in SNU719 and YCCEL1.

Supplemental Figure Legends mbio.00396-23-s0002.docx Legends for the supplemental figures.

Fig. S2 mbio.00396-23-s0003.tif Increased methylation at CpG sites found in CTCF binding region.

Fig. S3 mbio.00396-23-s0004.tif DCB treatment leads to an accumulation of intra and extracellular viral copies.

Fig. S4 mbio.00396-23-s0005.tif Epiallele changes at additional CTCF binding sites.

Fig. S5 mbio.00396-23-s0006.tif Epigenome of the BZLF1 locus.

ASM does not own the copyrights to Supplemental Material that may be linked to, or accessed through, an article. The authors have granted ASM a non-exclusive, world-wide license to publish the Supplemental Material files. Please contact the corresponding author directly for reuse.

## References

[1] Shibata D , Weiss LM . 1992. Epstein-Barr virus-associated gastric adenocarcinoma. Am J Pathol 140:769–774. 1314023 PMC1886378

[2] Rodriquenz MG , Roviello G , D’Angelo A , Lavacchi D , Roviello F , Polom K . 2020. MSI and EBV positive gastric cancer's subgroups and their link with novel immunotherapy. J Clin Med 9:1427. doi:10.3390/jcm9051427 32403403 PMC7291039

[3] Murphy G , Pfeiffer R , Camargo MC , Rabkin CS . 2009. Meta-analysis shows that prevalence of Epstein-Barr virus-positive gastric cancer differs based on sex and anatomic location. Gastroenterology 137:824–833. doi:10.1053/j.gastro.2009.05.001 19445939 PMC3513767

[4] Cancer Genome Atlas Research N . 2014. Comprehensive molecular characterization of gastric adenocarcinoma. Nature 513:202–209. doi:10.1038/nature13480 25079317 PMC4170219

[5] Zouridis H , Deng N , Ivanova T , Zhu Y , Wong B , Huang D , Wu YH , Wu Y , Tan IB , Liem N , Gopalakrishnan V , Luo Q , Wu J , Lee M , Yong WP , Goh LK , Teh BT , Rozen S , Tan P . 2012. Methylation subtypes and large-scale epigenetic alterations in gastric cancer. Sci Transl Med 4:156ra140. doi:10.1126/scitranslmed.3004504 23076357

[6] Fukayama M , Ushiku T . 2011. Epstein-Barr virus-associated gastric carcinoma. Pathol Res Pract 207:529–537. doi:10.1016/j.prp.2011.07.004 21944426

[7] Strong MJ , Laskow T , Nakhoul H , Blanchard E , Liu Y , Wang X , Baddoo M , Lin Z , Yin Q , Flemington EK . 2015. Latent expression of the Epstein-Barr virus (EBV)-encoded major histocompatibility complex class I TAP inhibitor, BNLF2A, in EBV-positive gastric carcinomas. J Virol 89:10110–10114. doi:10.1128/JVI.01110-15 26178981 PMC4577887

[8] Imai S , Koizumi S , Sugiura M , Tokunaga M , Uemura Y , Yamamoto N , Tanaka S , Sato E , Osato T . 1994. Gastric carcinoma: monoclonal epithelial malignant cells expressing Epstein-Barr virus latent infection protein. Proc Natl Acad Sci U S A 91:9131–9135. doi:10.1073/pnas.91.19.9131 8090780 PMC44761

[9] Sugiura M , Imai S , Tokunaga M , Koizumi S , Uchizawa M , Okamoto K , Osato T . 1996. Transcriptional analysis of Epstein-Barr virus gene expression in EBV-positive gastric carcinoma: unique viral latency in the tumour cells. Br J Cancer 74:625–631. doi:10.1038/bjc.1996.412 8761381 PMC2074674

[10] Wang A , Zhang W , Jin M , Zhang J , Li S , Tong F , Zhou Y . 2016. Differential expression of EBV proteins LMP1 and BHFR1 in EBV-associated gastric and nasopharyngeal cancer tissues. Mol Med Rep 13:4151–4158. doi:10.3892/mmr.2016.5087 27052804 PMC4838144

[11] Caruso LB , Guo R , Keith K , Madzo J , Maestri D , Boyle S , Wasserman J , Kossenkov A , Gewurz BE , Tempera I . 2022. The nuclear lamina binds the EBV genome during latency and regulates viral gene expression. PLoS Pathog 18:e1010400. doi:10.1371/journal.ppat.1010400 35421198 PMC9009669

[12] Morgan SM , Tanizawa H , Caruso LB , Hulse M , Kossenkov A , Madzo J , Keith K , Tan Y , Boyle S , Lieberman PM , Tempera I . 2022. The three-dimensional structure of Epstein-Barr virus genome varies by latency type and is regulated by PARP1 enzymatic activity. Nat Commun 13:187. doi:10.1038/s41467-021-27894-1 35039491 PMC8764100

[13] Tempera I , Wiedmer A , Dheekollu J , Lieberman PM . 2010. CTCF prevents the epigenetic drift of EBV latency promoter Qp. PLoS Pathog 6:e1001048. doi:10.1371/journal.ppat.1001048 20730088 PMC2921154

[14] Minarovits J . 2006. Epigenotypes of latent Herpesvirus genomes. Curr Top Microbiol Immunol 310:61–80. doi:10.1007/3-540-31181-5_5 16909907

[15] Keck KM , Moquin SA , He A , Fernandez SG , Somberg JJ , Liu SM , Martinez DM , Miranda JL . 2017. Bromodomain and extraterminal inhibitors block the Epstein-Barr virus lytic cycle at two distinct steps. J Biol Chem 292:13284–13295. doi:10.1074/jbc.M116.751644 28588024 PMC5555189

[16] Smith ZD , Meissner A . 2013. DNA methylation: roles in mammalian development. Nat Rev Genet 14:204–220. doi:10.1038/nrg3354 23400093

[17] Jones PA , Baylin SB . 2007. The epigenomics of cancer. Cell 128:683–692. doi:10.1016/j.cell.2007.01.029 17320506 PMC3894624

[18] Stanland LJ , Luftig MA . 2020. The role of EBV-induced hypermethylation in gastric cancer tumorigenesis. Viruses 12:1222. doi:10.3390/v12111222 33126718 PMC7693998

[19] Li L , Li C , Mao H , Du Z , Chan WY , Murray P , Luo B , Chan AT , Mok TS , Chan FK , Ambinder RF , Tao Q . 2016. Epigenetic inactivation of the CpG demethylase TET1 as a DNA methylation feedback loop in human cancers. Sci Rep 6:26591. doi:10.1038/srep26591 27225590 PMC4880909

[20] Falk KI , Szekely L , Aleman A , Ernberg I . 1998. Specific methylation patterns in two control regions of Epstein-Barr virus latency: the LMP-1-coding upstream regulatory region and an origin of DNA replication (oriP). J Virol 72:2969–2974. doi:10.1128/JVI.72.4.2969-2974.1998 9525618 PMC109743

[21] Robertson KD , Manns A , Swinnen LJ , Zong JC , Gulley ML , Ambinder RF . 1996. CpG methylation of the major Epstein-Barr virus latency promoter in Burkitt’s lymphoma and Hodgkin’s disease. Blood 88:3129–3136. 8874213

[22] Allday MJ , Kundu D , Finerty S , Griffin BE . 1990. CpG methylation of viral DNA in EBV-associated tumours. Int J Cancer 45:1125–1130. doi:10.1002/ijc.2910450623 2161800

[23] Robertson KD , Hayward SD , Ling PD , Samid D , Ambinder RF . 1995. Transcriptional activation of the Epstein-Barr virus latency C promoter after 5-azacytidine treatment: evidence that demethylation at a single CpG site is crucial. Mol Cell Biol 15:6150–6159. doi:10.1128/MCB.15.11.6150 7565767 PMC230866

[24] Masucci MG , Contreras-Salazar B , Ragnar E , Falk K , Minarovits J , Ernberg I , Klein G . 1989. 5-Azacytidine up regulates the expression of Epstein-Barr virus nuclear antigen 2 (EBNA-2) through EBNA-6 and latent membrane protein in the Burkitt’s lymphoma line rael. J Virol 63:3135–3141. doi:10.1128/JVI.63.7.3135-3141.1989 2470924 PMC250871

[25] Lupey-Green LN , Caruso LB , Madzo J , Martin KA , Tan Y , Hulse M , Tempera I . 2018. PARP1 stabilizes CTCF binding and chromatin structure to maintain Epstein-Barr virus latency type. J Virol 92:18. doi:10.1128/JVI.00755-18 PMC6146685 29976663

[26] Lee SH , Kim K-D , Cho M , Huh S , An SH , Seo D , Kang K , Lee M , Tanizawa H , Jung I , Cho H , Kang H . 2023. Characterization of a new CCCTC-binding factor binding site as a dual regulator of Epstein-Barr virus latent infection. PLoS Pathog 19:e1011078. doi:10.1371/journal.ppat.1011078 36696451 PMC9876287

[27] Dunn LEM , Lu F , Su C , Lieberman PM , Baines JD . 2023. Reactivation of Epstein-Barr virus from latency involves increased RNA polymerase activity at CTCF binding sites on the viral genome. J Virol 97:e0189422. doi:10.1128/jvi.01894-22 36744959 PMC9972995

[28] Chau CM , Zhang X-Y , McMahon SB , Lieberman PM . 2006. Regulation of Epstein-Barr virus latency type by the Chromatin boundary factor CTCF. J Virol 80:5723–5732. doi:10.1128/JVI.00025-06 16731911 PMC1472585

[29] Hashimoto H , Wang D , Horton JR , Zhang X , Corces VG , Cheng X . 2017. Structural basis for the versatile and methylation-dependent binding of CTCF to DNA. Mol Cell 66:711–720. doi:10.1016/j.molcel.2017.05.004 28529057 PMC5542067

[30] Wang H , Maurano MT , Qu H , Varley KE , Gertz J , Pauli F , Lee K , Canfield T , Weaver M , Sandstrom R , Thurman RE , Kaul R , Myers RM , Stamatoyannopoulos JA . 2012. Widespread plasticity in CTCF occupancy linked to DNA methylation. Genome Res 22:1680–1688. doi:10.1101/gr.136101.111 22955980 PMC3431485

[31] Nanavaty V , Abrash EW , Hong C , Park S , Fink EE , Li Z , Sweet TJ , Bhasin JM , Singuri S , Lee BH , Hwang TH , Ting AH . 2020. DNA methylation regulates alternative polyadenylation via CTCF and the Cohesin complex. Mol Cell 78:752–764. doi:10.1016/j.molcel.2020.03.024 32333838 PMC7245569

[32] Ohki I , Shimotake N , Fujita N , Jee J , Ikegami T , Nakao M , Shirakawa M . 2001. Solution structure of the methyl-CpG binding domain of human MBD1 in complex with methylated DNA. Cell 105:487–497. doi:10.1016/s0092-8674(01)00324-5 11371345

[33] Hofmeister BT , Lee K , Rohr NA , Hall DW , Schmitz RJ . 2017. Stable inheritance of DNA methylation allows creation of epigenotype maps and the study of epiallele inheritance patterns in the absence of genetic variation. Genome Biol 18:155. doi:10.1186/s13059-017-1288-x 28814343 PMC5559844

[34] Shuto T , Nishikawa J , Shimokuri K , Yanagi A , Takagi T , Takagi F , Miura O , Iida M , Nagano H , Takemoto Y , Harada E , Suehiro Y , Yamasaki T , Okamoto T , Sakaida I . 2019. Establishment of a screening method for Epstein-Barr virus-associated gastric carcinoma by droplet digital PCR. Microorganisms 7:12. doi:10.3390/microorganisms7120628 31795435 PMC6956032

[35] Li L , Su X , Choi GCG , Cao Y , Ambinder RF , Tao Q . 2012. Methylation profiling of Epstein-Barr virus immediate-early gene promoters, BZLF1 and BRLF1 in tumors of epithelial, NK- and B-cell origins. BMC Cancer 12:125. doi:10.1186/1471-2407-12-125 22458933 PMC3362778

[36] Gu H , Smith ZD , Bock C , Boyle P , Gnirke A , Meissner A . 2011. Preparation of reduced representation bisulfite sequencing libraries for genome-scale DNA methylation profiling. Nat Protoc 6:468–481. doi:10.1038/nprot.2010.190 21412275

[37] Moll P , Ante M , Seitz A , Reda T . 2014. Quantseq 3′ mRNA sequencing for RNA quantification. Nat Methods 11:i–iii. doi:10.1038/nmeth.f.376

[38] Li H , Durbin R . 2009. Fast and accurate short read alignment with burrows-wheeler transform. Bioinformatics 25:1754–1760. doi:10.1093/bioinformatics/btp324 19451168 PMC2705234

[39] Ramírez F , Dündar F , Diehl S , Grüning BA , Manke T . 2014. Deeptools: a flexible platform for exploring deep-sequencing data. Nucleic Acids Res 42:W187–W191. doi:10.1093/nar/gku365 24799436 PMC4086134

[40] Dobin A , Davis CA , Schlesinger F , Drenkow J , Zaleski C , Jha S , Batut P , Chaisson M , Gingeras TR . 2013. STAR: ultrafast universal RNA-seq aligner. Bioinformatics 29:15–21. doi:10.1093/bioinformatics/bts635 23104886 PMC3530905

[41] Li B , Dewey CN . 2011. RSEM: accurate transcript quantification from RNA-Seq data with or without a reference genome. BMC Bioinformatics 12:323. doi:10.1186/1471-2105-12-323 21816040 PMC3163565

[42] Love MI , Huber W , Anders S . 2014. Moderated estimation of fold change and dispersion for RNA-Seq data with DESeq2. Genome Biol 15:550. doi:10.1186/s13059-014-0550-8 25516281 PMC4302049

[43] Langmead B , Salzberg SL . 2012. Fast gapped-read alignment with bowtie 2. Nat Methods 9:357–359. doi:10.1038/nmeth.1923 22388286 PMC3322381

[44] Heinz S , Benner C , Spann N , Bertolino E , Lin YC , Laslo P , Cheng JX , Murre C , Singh H , Glass CK . 2010. Simple combinations of lineage-determining transcription factors prime cis-regulatory elements required for macrophage and B cell identities. Mol Cell 38:576–589. doi:10.1016/j.molcel.2010.05.004 20513432 PMC2898526

[45] Kent WJ , Zweig AS , Barber G , Hinrichs AS , Karolchik D . 2010. BigWig and BigBed: enabling browsing of large distributed Datasets. Bioinformatics 26:2204–2207. doi:10.1093/bioinformatics/btq351 20639541 PMC2922891

[46] Lin C-T , Leibovitch EC , Almira-Suarez MI , Jacobson S . 2016. Human herpesvirus multiplex ddPCR detection in brain tissue from low- and high-grade astrocytoma cases and controls. Infect Agent Cancer 11:32. doi:10.1186/s13027-016-0081-x 27462365 PMC4960850

[47] Oh ST , Seo JS , Moon UY , Kang KH , Shin D-J , Yoon SK , Kim WH , Park J-G , Lee SK . 2004. A naturally derived gastric cancer cell line shows latency I Epstein-Barr virus infection closely resembling EBV-associated gastric cancer. Virology 320:330–336. doi:10.1016/j.virol.2003.12.005 15016554

[48] Kim DN , Seo MK , Choi H , Kim SY , Shin HJ , Yoon A-R , Tao Q , Rha SY , Lee SK . 2013. Characterization of naturally Epstein-Barr virus-infected gastric carcinoma cell line YCCEL1. J Gen Virol 94:497–506. doi:10.1099/vir.0.045237-0 23175241

[49] Rowe M , Lear AL , Croom-Carter D , Davies AH , Rickinson AB . 1992. Three pathways of Epstein-Barr virus gene activation from EBNA1-positive latency in B lymphocytes. J Virol 66:122–131. doi:10.1128/JVI.66.1.122-131.1992 1309242 PMC238267

[50] Rowe DT , Hall L , Joab I , Laux G . 1990. Identification of the Epstein-Barr virus terminal protein gene products in latently infected lymphocytes. J Virol 64:2866–2875. doi:10.1128/JVI.64.6.2866-2875.1990 2159547 PMC249469

[51] Weigel R , Fischer DK , Heston L , Miller G . 1985. Constitutive expression of Epstein-Barr virus-encoded RNAs and nuclear antigen during latency and after induction of Epstein-Barr virus replication. J Virol 53:254–259. doi:10.1128/JVI.53.1.254-259.1985 2981344 PMC255024

[52] Yetming KD , Lupey-Green LN , Biryukov S , Hughes DJ , Marendy EM , Miranda JL , Sample JT . 2020. The BHLF1 locus of Epstein-Barr virus contributes to viral latency and B-cell immortalization. J Virol 94:17. doi:10.1128/JVI.01215-20 PMC7431786 32581094

[53] Guo R , Jiang C , Zhang Y , Govande A , Trudeau SJ , Chen F , Fry CJ , Puri R , Wolinsky E , Schineller M , Frost TC , Gebre M , Zhao B , Giulino-Roth L , Doench JG , Teng M , Gewurz BE . 2020. MYC controls the Epstein-Barr virus lytic switch. Mol Cell 78:653–669. doi:10.1016/j.molcel.2020.03.025 32315601 PMC7245572

[54] Holdorf MM , Cooper SB , Yamamoto KR , Miranda JJL . 2011. Occupancy of chromatin organizers in the Epstein-Barr virus genome. Virology 415:1–5. doi:10.1016/j.virol.2011.04.004 21550623 PMC3808970

[55] Chen H-S , Martin KA , Lu F , Lupey LN , Mueller JM , Lieberman PM , Tempera I . 2014. Epigenetic deregulation of the LMP1/LMP2 locus of Epstein-Barr virus by mutation of a single CTCF-cohesin binding site. J Virol 88:1703–1713. doi:10.1128/JVI.02209-13 24257606 PMC3911611

[56] Bergbauer M , Kalla M , Schmeinck A , Göbel C , Rothbauer U , Eck S , Benet-Pagès A , Strom TM , Hammerschmidt W . 2010. CpG-methylation regulates a class of Epstein-Barr virus promoters. PLoS Pathog 6:e1001114. doi:10.1371/journal.ppat.1001114 20886097 PMC2944802

[57] Dickerson SJ , Xing Y , Robinson AR , Seaman WT , Gruffat H , Kenney SC . 2009. Methylation-dependent binding of the Epstein-Barr virus BZLF1 protein to viral promoters. PLoS Pathog 5:e1000356. doi:10.1371/journal.ppat.1000356 19325883 PMC2654727

[58] Karlsson QH , Schelcher C , Verrall E , Petosa C , Sinclair AJ . 2008. Methylated DNA recognition during the reversal of epigenetic silencing is regulated by cysteine and serine residues in the Epstein-Barr virus lytic switch protein. PLoS Pathog 4:e1000005. doi:10.1371/journal.ppat.1000005 18369464 PMC2267006

[59] Schaeffner M , Mrozek-Gorska P , Buschle A , Woellmer A , Tagawa T , Cernilogar FM , Schotta G , Krietenstein N , Lieleg C , Korber P , Hammerschmidt W . 2019. BZLF1 interacts with chromatin remodelers promoting escape from latent infections with EBV. Life Sci Alliance 2:e201800108. doi:10.26508/lsa.201800108 30926617 PMC6441497

[60] Ali A , Ohashi M , Casco A , Djavadian R , Eichelberg M , Kenney SC , Johannsen E . 2022. Rta is the principal activator of Epstein-Barr virus epithelial lytic transcription. PLoS Pathog 18:e1010886. doi:10.1371/journal.ppat.1010886 36174106 PMC9553042

