# Supplementary figures and images for: Decitabine disrupts EBV genomic epiallele DNA methylation patterns around CTCF binding sites to increase chromatin accessibility and lytic transcription in gastric cancer
Source: mBio. 2023 Aug 22;14(5):e00396-23. doi: 10.1128/mbio.00396-23 (PMC10653948; doi:10.1128/mbio.00396-23)

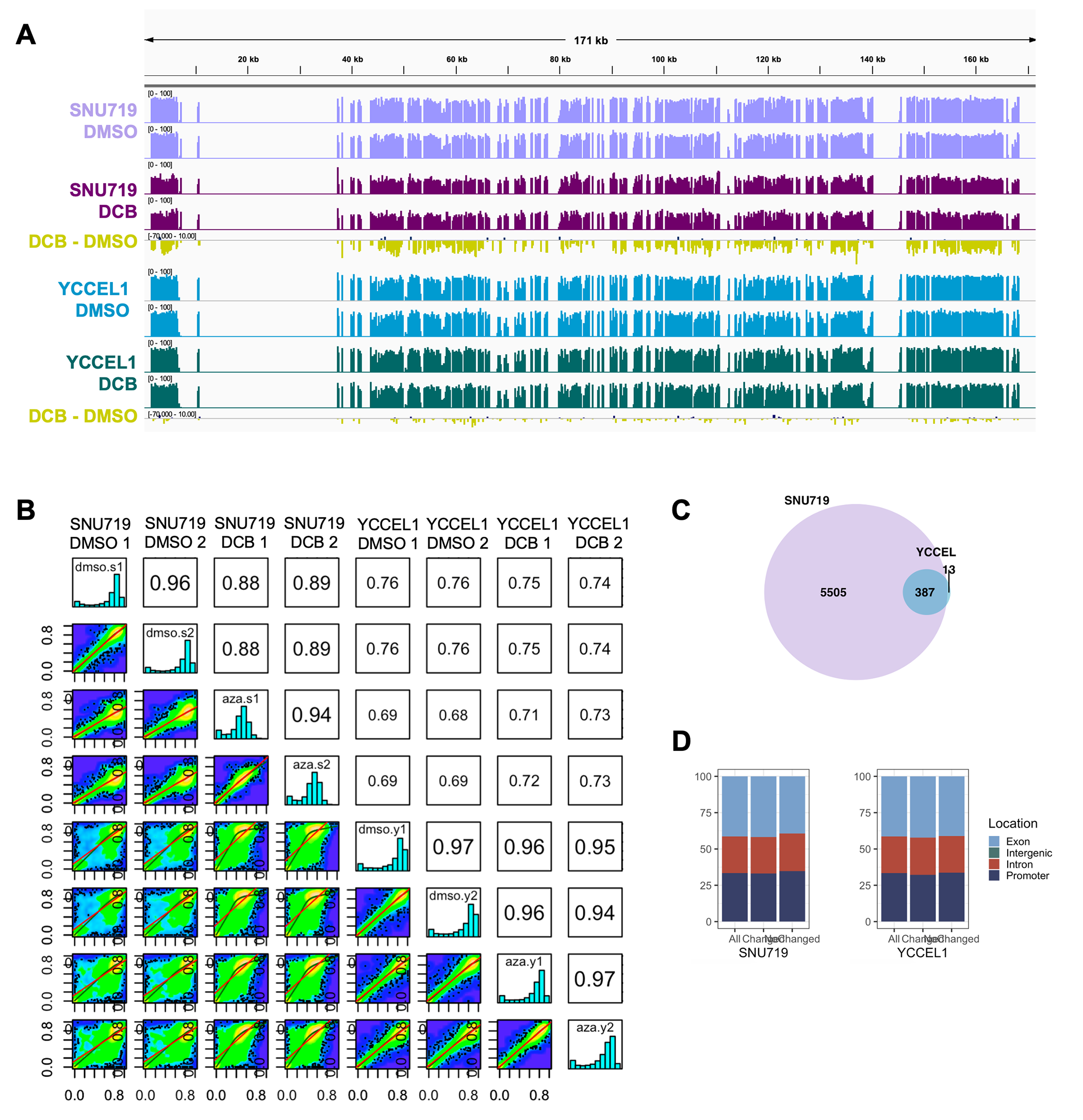

Supplement: Fig. S1 — Conserved response to DCB in SNU719 and YCCEL1. [file mbio.00396-23-s0001.tif]

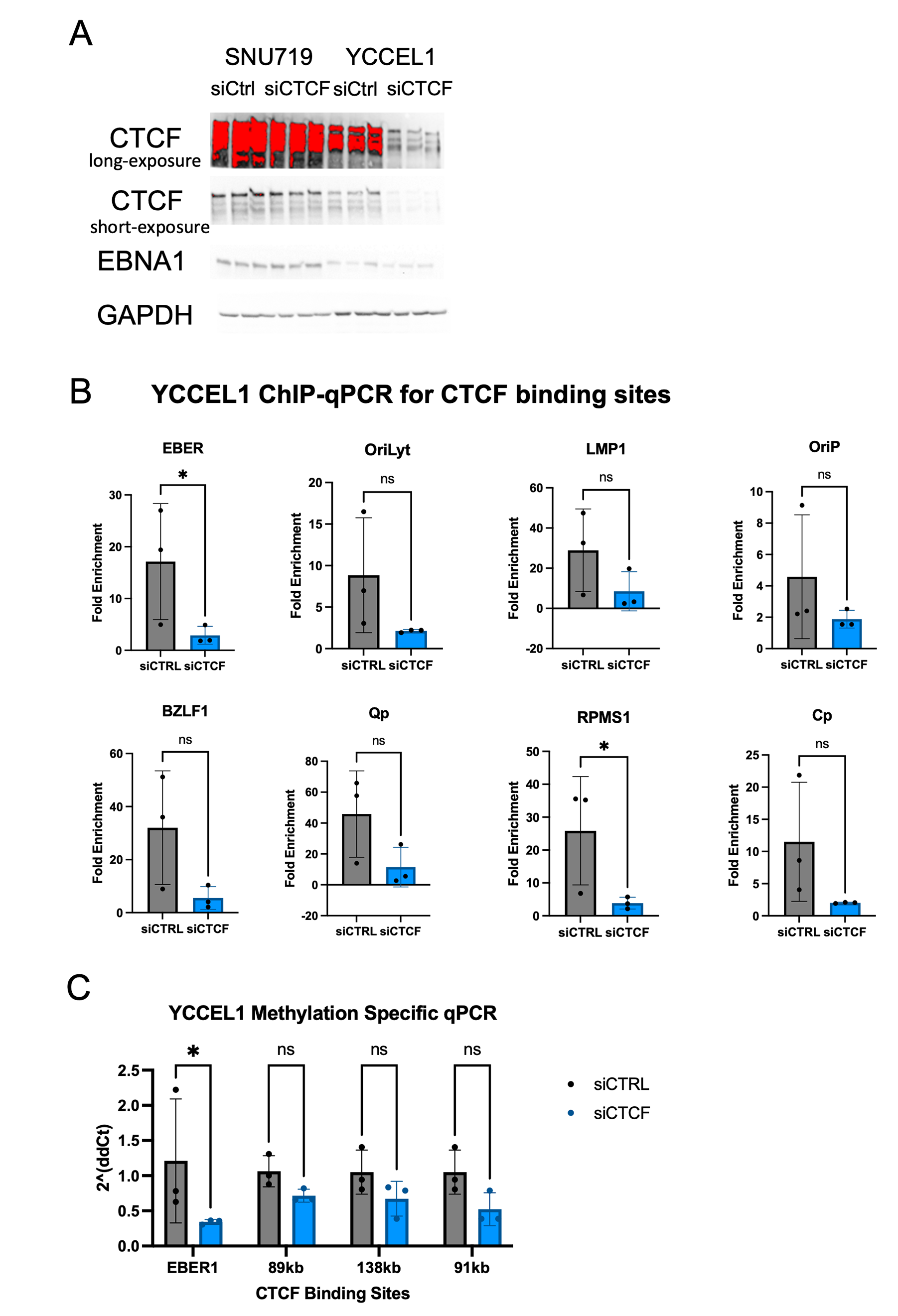

Supplement: Fig. S2 — Increased methylation at CpG sites found in CTCF binding region. [file mbio.00396-23-s0003.tif]

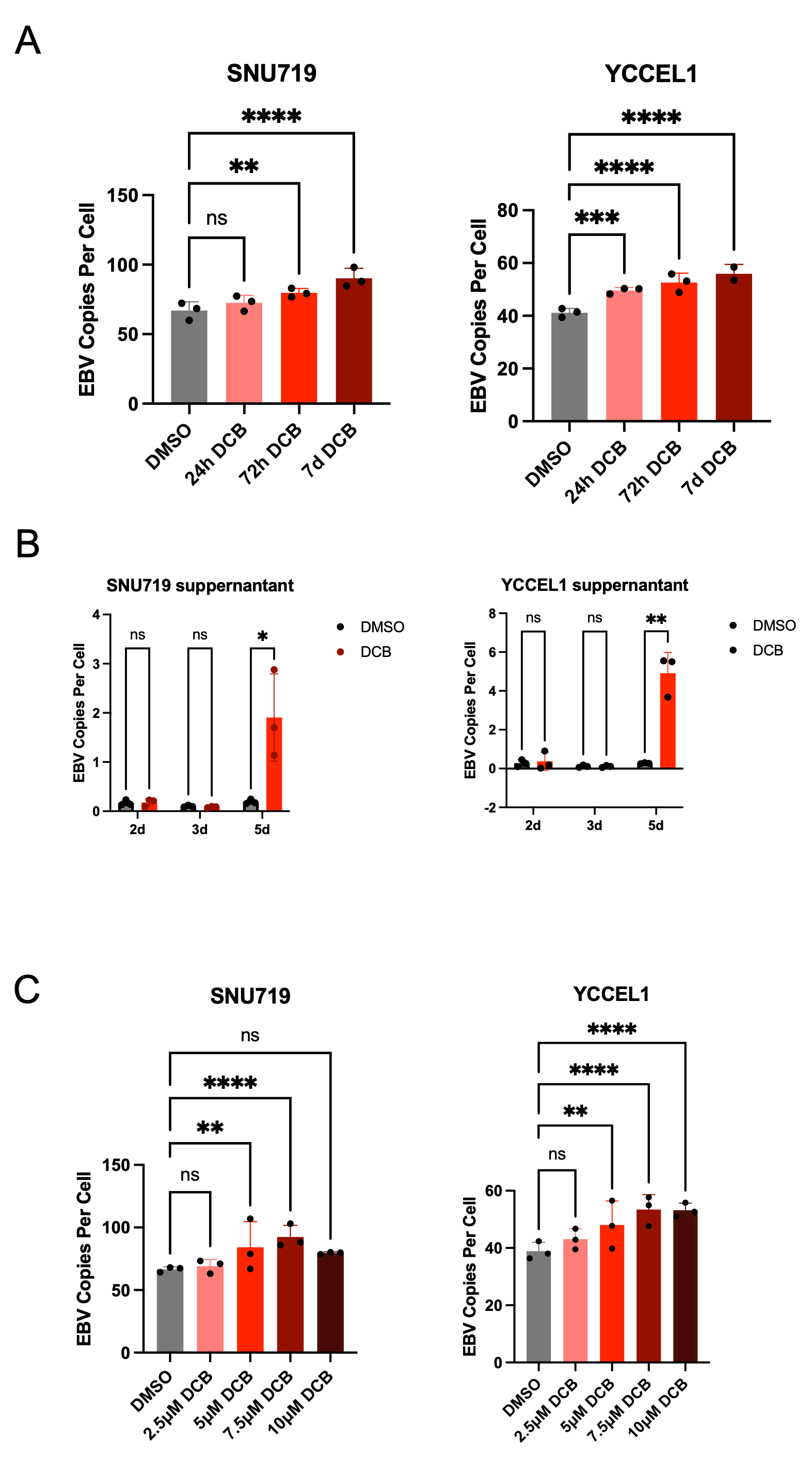

Supplement: Fig. S3 — DCB treatment leads to an accumulation of intra and extracellular viral copies. [file mbio.00396-23-s0004.tif]

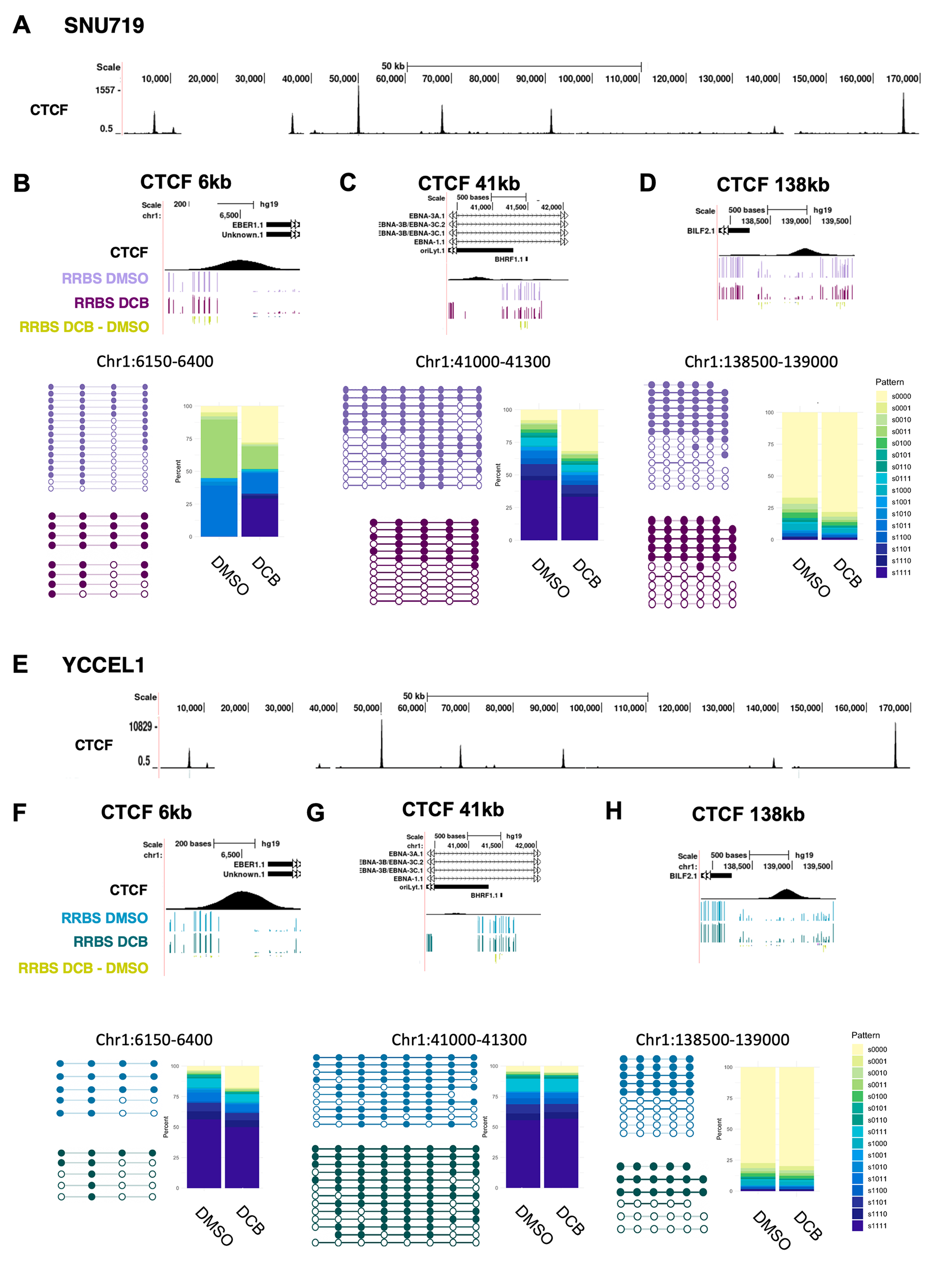

Supplement: Fig. S4 — Epiallele changes at additional CTCF binding sites. [file mbio.00396-23-s0005.tif]

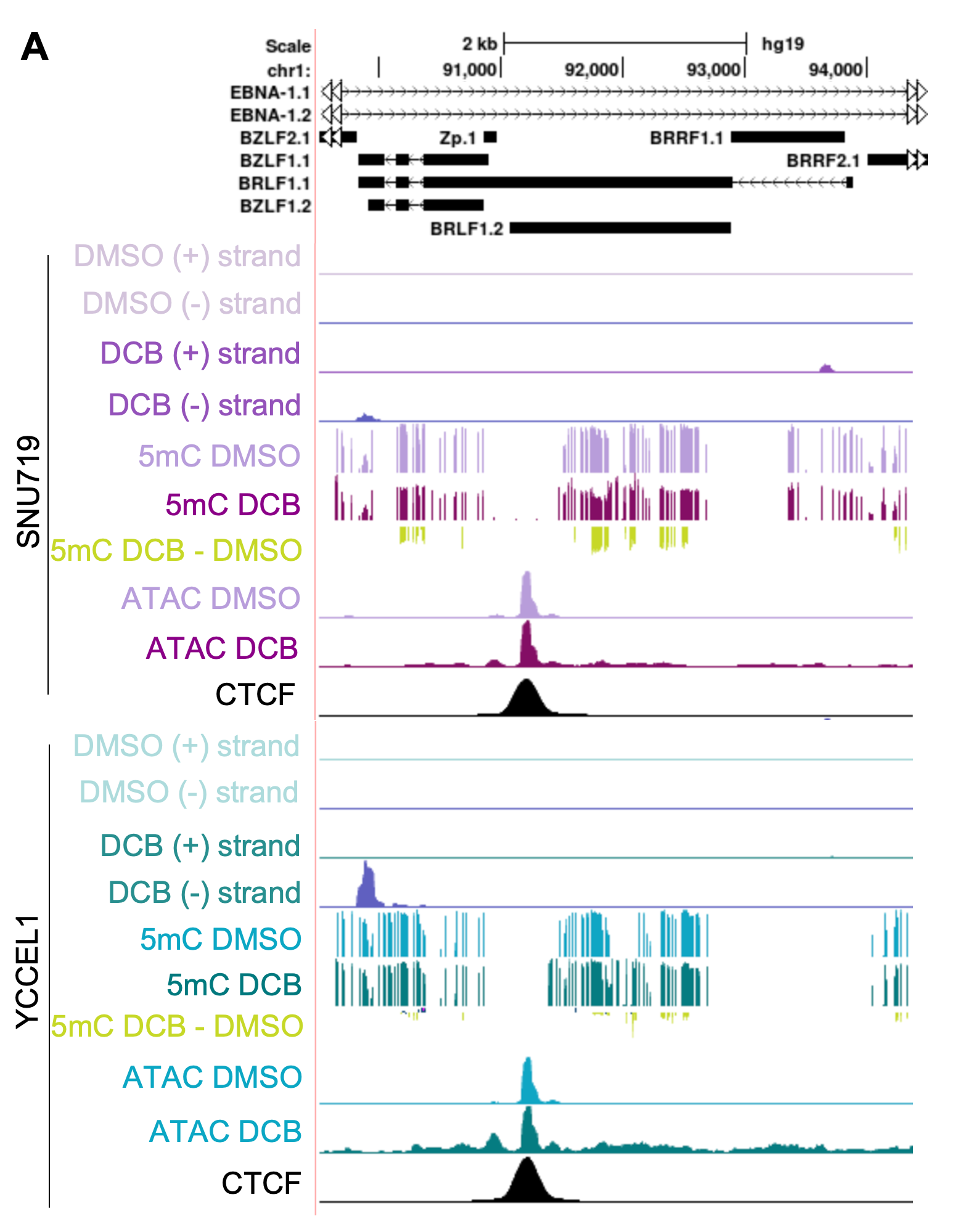

Supplement: Fig. S5 — Epigenome of the BZLF1 locus. [file mbio.00396-23-s0006.tif]
