# Supplementary material for: Decitabine disrupts EBV genomic epiallele DNA methylation patterns around CTCF binding sites to increase chromatin accessibility and lytic transcription in gastric cancer
Source: mBio. 2023 Aug 22;14(5):e00396-23. doi: 10.1128/mbio.00396-23 (PMC10653948; doi:10.1128/mbio.00396-23)
Supplement: Supplemental Figure Legends — Legends for the supplemental figures. [file mbio.00396-23-s0002.docx]

**Supplemental Figure 1: Conserved response to DCB in SNU719 and YCCEL1.**

A) EBV genome tracks for all RRBS duplicates for DMSO and DCB treated samples in SNU719 and YCCEL1. B) A correlogram between all samples. The top half displays the Pearson correlation coefficient. Down the middle is a histogram for the CpG distribution for each sample from 0 to 100% methylated. The bottom half displays a density plot of the correlation between two samples based on 5mC distribution. C) Venn diagram of significantly changed CpG sites. D) Stacked bar plot showing the distribution of CpG sites in exons, introns, promoters, and intergenic regions. Shown are all detectable CpG sites, CpG sites that are significantly changes, and CpG sites that do not change.

**Supplemental Figure 2: Increased methylation at CpG sites found in CTCF binding regions.**

A) Western blot of siCTCF knockdown in SNU719 and YCCEL1. B) CTCF ChIP-qPCR for CTCF binding regions after siCTCF knockdown in YCCEL1 cells. C) Methylation specific qPCR of bisulfite converted DNA for CpG sites found in four different CTCF binding regions after siCTCF knockdown.

**Supplemental Figure 3: DCB treatment leads to an accumulation of intra and extracellular viral copies.**

A) Digital droplet PCR (ddPCR) quantifying the EBV copy number per cell after 7.5uM DCB treatment. B) ddPCR quantification of EBV copy number in the supernatant. C) ddPCR quantification after 3d treatment of cellular EBV copy number at varying DCB concentrations.

**Supplemental Figure 4: Epiallele changes at additional CTCF binding sites.**

A) EBV genome track for CTCF binding sites identified by ChIP-seq in SNU719. 5mC epiallele patterns (representative sample) around B) CTCF 6kb C) CTCF 41kb D) CTCF 138kb binding sites for DMSO and DCB treatment with a stacked bar plot quantify percent distribution of epialleles. Methylation patterning is represented by 1 for a methylated CpG or 0 for a unmethylated CpG. E) EBV genomic track for CTCF ChIP-seq for YCCEL1 5mC epiallele patterns (representative sample) around F) CTCF 6kb G) CTCF 41kb H) CTCF 138kb binding sites for DMSO and DCB treatment with a stacked bar plot quantifying percent distribution of epialleles.

**Supplemental Figure 5: Epigenome of the BZLF1 locus.**

A) EBV genome track for CTCF binding sites identified by ChIP-seq in SNU719 (top) and YCCEL1 (bottom) for forward and reverse strand reads from RNA-seq, 5mC from RRBS, ATAC-seq, and CTCF ChIP-seq sites.
